# Genetic–Epigenetic Interplay in Epilepsy: Pathways, Biomarkers, and Epigenome-Targeted Therapies

**DOI:** 10.3390/epigenomes10010010

**Published:** 2026-02-10

**Authors:** Andra-Giorgiana Zaruha, Patricia Codreanu, Mădălin-Codruț Coman, Monica Andreea Novac II, Simona Gabriela Duță-Ion, Ioana Ruxandra Jugănaru, Iulian Andrei Hotinceanu, Andra Dan, Livia Mălina Burtavel, Anca-Elena Eftenoiu, Diana Bârcă, Andreea Ionescu, Cerasela Paraschiv, Viorica-Elena Rădoi

**Affiliations:** 1 Department of Medical Genetics, “Carol Davila” University of Medicine and Pharmacy, 020021 Bucharest, Romania; andra-giorgiana.zaruha@rez.umfcd.ro (A.-G.Z.); patricia-christina.codreanu@rez.umfcd.ro (P.C.); simona-gabriela.duta@rez.umfcd.ro (S.G.D.-I.); ioana-ruxandra.calapod@rez.umfcd.ro (I.R.J.); iulian.hotinceanu@s.unibuc.ro (I.A.H.); andra.dan@rez.umfcd.ro (A.D.); livia-malina.burtavel@rez.umfcd.ro (L.M.B.); anca-elena.eftenoiu@rez.umfcd.ro (A.-E.E.); cerasela.paraschiv@umfcd.ro (C.P.); viorica.radoi@umfcd.ro (V.-E.R.); 2 Pediatric Neurology Department, Prof. Dr. Alex. Obregia Clinical Hospital of Psychiatry, 041914 Bucharest, Romania; diana.barca@umfcd.ro; 3 Pediatric Neurology Department, “Carol Davila” University of Medicine and Pharmacy, 020021 Bucharest, Romania; 4 Department of Genetics, Faculty of Biology, University of Bucharest, 050095 Bucharest, Romania; andreea_carmen_ionescu@yahoo.com; 5 “Alessandrescu-Rusescu” National Institute for Maternal and Child Health, 20382 Bucharest, Romania

**Keywords:** epilepsy, epigenetics, chromatin remodeling, histone modifications, DNA methylation, non-coding RNA, precision medicine, CRISPR, antisense oligonucleotides

## Abstract

Epilepsy is a heterogeneous neurological disorder with a strong genetic basis, yet recent evidence underscores the critical role of epigenetic mechanisms in its pathogenesis. This review synthesizes current knowledge on how chromatin remodeling, histone modifications, DNA methylation, and transcriptional regulation intersect with classical channelopathies and signaling pathways. We emphasize how epigenetic dysregulation contributes to neuronal excitability and network plasticity, particularly through interactions with mTOR, PI3K-AKT, and GABAergic signaling cascades. The convergence of genetic mutations and epigenetic modifications creates a dynamic landscape in which environmental factors can modify gene expression and contribute to the development of epilepsy. Emerging therapeutic strategies—including epigenetic drugs (HDAC inhibitors, DNMT inhibitors), CRISPR/dCas9-based epigenome editing, and multi-omics approaches—offer promising avenues for precision medicine. This review provides a comprehensive synthesis of genetic and epigenetic mechanisms in epilepsy, examining how these layers interact to produce disease phenotypes and discussing the therapeutic implications of this multilayered regulation.

## 1. Introduction

Epilepsy affects approximately 1% of the global population, making it one of the most common neurological diseases worldwide, and encompasses diverse seizure types and clinical presentations. A significant proportion of epilepsy patients, approximately 30%, will eventually progress to chronic drug-resistant epilepsy, indicating a lack of response to currently available anti-seizure medications. The chronic and recurrent nature of epileptic seizures, coupled with associated mortality and substantial economic burden, profoundly impacts quality of life. Epilepsy is a chronic neurological disorder characterized by a complex interplay of genetic and environmental factors that disrupts the normal electrical activity of the central nervous system, leading to recurrent seizures [1]. The International League Against Epilepsy (ILAE) classifies epilepsies by seizure type, epilepsy type, and syndromes based on age of onset and etiology [2]. Genetic predisposition is well-established, with estimates suggesting genetic contribution in 70–80% of cases [3]. However, traditional genetic mechanisms alone are insufficient to fully elucidate epilepsy pathogenesis. Familial linkage analyses have identified genes explaining less than 1% of cases, with the majority exhibiting complex genetic inheritance patterns. This gap between genetic findings and clinical reality highlights the critical importance of epigenetic regulation in epilepsy research.

## 2. Materials and Methods

A comprehensive literature review was conducted to identify relevant publications on molecular pathways, epigenetic mechanisms, and emerging therapies for genetic epilepsy syndromes.

Search strategy: The databases used were PubMed, Scopus, and Web of Science. Articles published between 2010 and 2025 were prioritized to capture the most recent developments, although seminal older studies were also included where relevant. Search terms included “genetic epilepsy syndromes”, “epigenetics in epilepsy”, “molecular pathways”, “targeted therapy”, “ion channel mutations”, “SCN1A mutations”, “GABAergic signaling”, “mTOR pathway”, “DNA methylation epilepsy”, “histone modifications epilepsy”, “non-coding RNA epilepsy”, “epigenetic regulation”, “CRISPR epilepsy” and “antisense oligonucleotides.”

Inclusion criteria:

- Peer-reviewed studies, clinical trials, and high-impact review articles;
- Reports from regulatory bodies (FDA, EMA) on therapeutic agent approval;
- English-language publications only;
- Focus on molecular mechanisms, therapeutic interventions, and clinical efficacy.

Data extraction: The focus was on identifying key molecular mechanisms, epigenetic changes, therapeutic interventions, and their clinical efficacy. Systematic reviews and meta-analyses were referenced to ensure breadth and reliability of conclusions.

## 3. Review of Evidence

### 3.1. Genetic Causes and Molecular Pathways in Epilepsy

Genetic epilepsies arise from mutations in genes that play critical roles in brain development, neuronal signaling, and ion channel function. These mutations disrupt normal brain function through several mechanisms:

Brain development: Genetic mutations can result in structural anomalies including altered neuronal connectivity or cortical developmental malformations, increasing epilepsy susceptibility.

Neuronal signaling: Genes regulating neuronal signaling maintain the balance between excitation and inhibition. Mutations cause imbalances leading to excessive neuronal firing. Examples include genes encoding neurotransmitter receptors or metabolic enzymes.

Ion channel function: Ion channel genes regulate ion movement across neuronal membranes, which is vital for electrical signal initiation and propagation. Mutations affecting sodium, potassium, and calcium channels are particularly significant.

From genetic and phenotypic perspectives, epilepsies are highly diverse, with pathogenic variants including nonsense, missense, splice-site mutations, and small deletions or insertions.

### 3.2. The Epigenetic Paradigm in Epilepsy

Epigenetic processes translate information from short-lived cellular signals and changes in neuronal activity into lasting effects on gene expression. Key molecular mediators include DNA methylation, histone modifications, chromatin remodeling, and noncoding RNAs. These mechanisms provide a dynamic layer of gene expression control that bridges genetic predisposition and environmental influences.

The process of epileptogenesis is characterized by widespread changes in gene expression—both activation and silencing—that affect neuronal death, gliosis, neuroinflammation, ion channels, neurotransmitter receptors, and network-level remodeling. Transcription is strongly influenced by chromatin state—the nucleosome assembly that is the basic unit of DNA packaging. Epigenetics encompasses processes that influence gene transcription by altering chromatin state and that persist long after the initial stimulus has ceased.

Aberrant gene expression disrupts gene networks that regulate inflammation, gliosis, synaptic structure, and neuronal function. Although multiple causative genes have been implicated, the replicability of findings is low, and functional validation is limited. Epigenetic regulation has emerged as a pivotal focus, contributing significantly to understanding the intricate mechanisms underlying epilepsy.

### 3.3. Molecular Pathways in Genetic Epilepsy

Genetic epilepsy syndromes arise from a convergence of disrupted molecular signaling pathways that compromise neuronal stability and excitability. Rather than viewing these mutations in isolation, a systems-level approach reveals how distinct pathways—ranging from ion channel dynamics to epigenetic regulation—interact to shape the epileptic phenotype. This framework not only clarifies pathophysiological mechanisms but also highlights therapeutic opportunities and unresolved questions [4].

**A.**  Ion channel signaling pathways—the foundation of hyperexcitability

**Figure 1:**
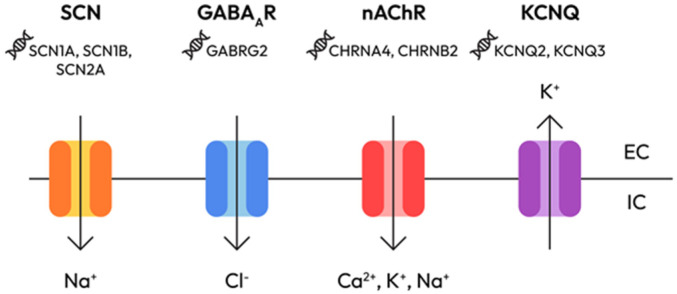
Normal Activity of Key Neuronal Membrane Channels and Receptors. GABA_A_R = gamma-aminobutyric acid type A receptor; EC = extracellular; IC = intracellular; nAChR = nicotinic acetylcholine receptor; KCNQ = a family of voltage-gated K+ channels; SCN = sodium channel. This figure illustrates the functions of major neuronal ion channels and receptors, emphasizing the key genes that encode their components and are implicated in genetic epilepsies.

Ion channels (Figure 1) are central to neuronal excitability and mutations in their encoding genes represent a primary mechanism in genetic epilepsies [5]. Dysfunctions of mutant ion channels, whether voltage-dependent or ligand-gated, are a major cause of rare monogenic idiopathic epilepsies and are also suspected to play a significant role in more common forms of epilepsy, such as juvenile myoclonic epilepsy and both infantile and juvenile absence epilepsies [6].

Sodium channels (SCN1A, SCN2A, SCN8A) can produce either gain-of-function (GOF) or loss-of-function (LOF) effects [7]:

- SCN1A loss-of-function in interneurons causes Dravet syndrome via GABAergic disinhibition;
- SCN2A gain-of-function leads to early-onset seizures, while loss-of-function results in autism with minimal epilepsy;
- SCN8A gain-of-function causes high-frequency, intractable seizures through enhanced persistent sodium current.

Potassium channels (KCNQ2/3, KCNA1/2, KCNH1) are critical for repolarization and afterhyperpolarization. Mutations in these channels cause prolonged depolarization and increased neuronal excitability [8].

- KCNQ2/3 loss-of-function reduces M-current, causing neonatal epileptic encephalopathy.
- KCNA2 gain-of-function associates with cerebellar ataxia and epilepsy.
- KCNH1 gain-of-function mutations are described in Temple–Baraitser and Zimmermann–Laband syndromes; KCNH1 mosaicism explains phenotypic variability, underscoring the importance of somatic mutation load in epileptogenesis.
- KCTD7 mutations linked to progressive myoclonic epilepsy type 3, involving modulation of inward-rectifier K+ currents [9].

Recently, Kv1.1 channels (encoded by KCNA1) have been engineered to enhance repolarization and suppress seizures in in vivo models—paving the way for ion channel gene therapy [10].

Other channels:

- Voltage-gated calcium channels (CACNA1A, CACNA1H) and glutamate receptors modulate neurotransmitter release and synaptic plasticity. Dysregulation contributes to cortical hyperexcitability [11].
- Hyperpolarization-Activated Cyclic Nucleotide-Gated Channel (HCN1/2) loss-of-function increases neuronal excitability and seizure susceptibility [12].
- Chloride channel (CLCN2) and GABA_A receptor subunits (GABRA1, GABRB3, GABRG2) maintain inhibitory tone. Mutations often lead to a shift in chloride reversal potential, weakening GABAergic inhibition [13].

Despite extensive characterization, genotype–phenotype correlations remain incomplete, especially regarding variable expressivity and treatment response. A novel insight into epileptogenesis is the concept of degeneracy in channel function; the concept of degeneracy in channel function refers to the idea that multiple molecular pathways can produce the same functional output [14]. In epilepsy, distinct channel mutations can result in convergent seizure phenotypes. Systems biology modeling shows that epileptic networks adapt by recruiting alternative ion channels when one is dysfunctional. This complicates monogenic therapeutic targeting, necessitating combinatorial or network-aware strategies.

Some of the therapeutic implications of ion channels are depicted in Table 1.

**B.**  mTOR and PI3K-AKT signaling pathways—growth pathways in epileptogenesis

**Figure 2:**
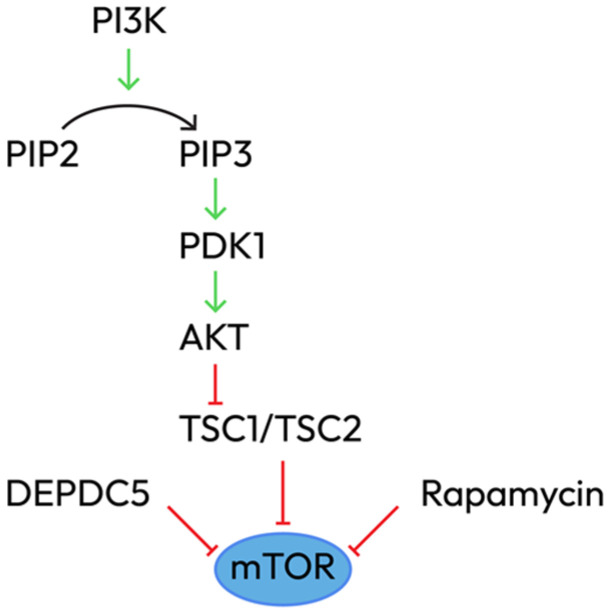
mTOR and PI3K-AKT Signaling Pathways in Epilepsy. Activation is represented by green arrows, and inhibition by red bars. The name mTOR reflects its inhibition by rapamycin, a macrolide compound.

The mTOR pathway governs cellular growth and synaptic plasticity. Dysregulation leads to abnormal brain development and epilepsy [19]. mTOR exists in two complexes: mTORC1 (regulates protein synthesis, autophagy, and cell growth) and mTORC2 (cytoskeletal organization and cell survival). Upstream regulators include TSC1/TSC2 (GTPase-activating proteins that inhibit mTORC1 via RHEB) and DEPDC5 (a component of the GATOR1 complex that senses amino acid availability) (Figure 2).

Pathogenic mechanisms start with mutations in TSC1, TSC2, MTOR, DEPDC5, NPRL2, and NPRL3, which lead to constitutive mTORC1 activation, promoting aberrant neuronal growth and differentiation, cortical lamination defects, glioneuronal proliferation, and synaptic imbalances (increased excitatory and decreased inhibitory synapse formation).

Somatic MTOR mutations (e.g., p.Ser2215Phe) were identified in focal cortical dysplasia (FCD) type IIb lesions, correlating with seizure focus and balloon cell presence [20].

Autophagy crosstalks with mTOR in epilepsy. In neurons, autophagy regulates synaptic protein turnover, dendritic spine remodeling, and mitochondrial quality control. mTORC1 hyperactivation suppresses autophagy, leading to dysfunctional organelle accumulation and hyperexcitability. In experimental models, mice with neuron-specific deletion of Atg7 exhibit spontaneous seizures, highlighting the necessity of intact autophagic flux for seizure suppression [21].

The role of DEPDC5 mutations in epilepsy remains a subject of ongoing debate, reflecting the broader complexity of genotype–phenotype relationships in genetic epilepsy. Although numerous studies have linked DEPDC5 loss-of-function variants to focal epilepsies and dysregulation of the mTOR pathway, the evidence remains far from uniform. Notably, animal models have shown that heterozygous DEPDC5 knockout does not consistently lead to seizures, suggesting that the mutation alone may be insufficient to trigger the epileptic phenotype. This raises important questions about the influence of additional genetic modifiers, environmental triggers, or epigenetic factors that may modulate disease expression. Clinically, the variability in seizure severity and onset—even among individuals carrying the same mutation—further complicates our understanding. These inconsistencies highlight a critical gap in current research: the need to move beyond single-gene associations toward a more integrated model that considers network-level interactions, compensatory and epigenetic mechanisms.

Somatic mosaicism was described in connection with epileptogenesis. Many focal epilepsies, especially FCD type II, are now understood as somatic mosaic disorders, involving low-frequency pathogenic variants in the mTOR pathway, which require ultra-deep sequencing (>500×) for detection [22]. Layer V pyramidal neurons are particularly vulnerable to mTOR overactivation [23]. mTOR mutations may alter chromatin accessibility and transcription factor dynamics [24], thereby altering excitability-related gene expression and contributing to disease.

Clinical and therapeutic implications of mTOR pathways are depicted in Table 2.

Therapeutic modulation of autophagy uses Sirolimus and Everolimus, mTOR inhibitors that can restore autophagic flux and have shown efficacy in TSC-related epilepsy. Emerging molecules (e.g., ULK1 agonists, AMPK activators) are under investigation for autophagy restoration without full mTORC1 inhibition [21].

While mTOR inhibitors have shown promise, the long-term impact on neurodevelopment and seizure control warrants further investigation.

**C.**  GABAergic signaling pathway—inhibitory tone and network modulation

**Figure 3:**
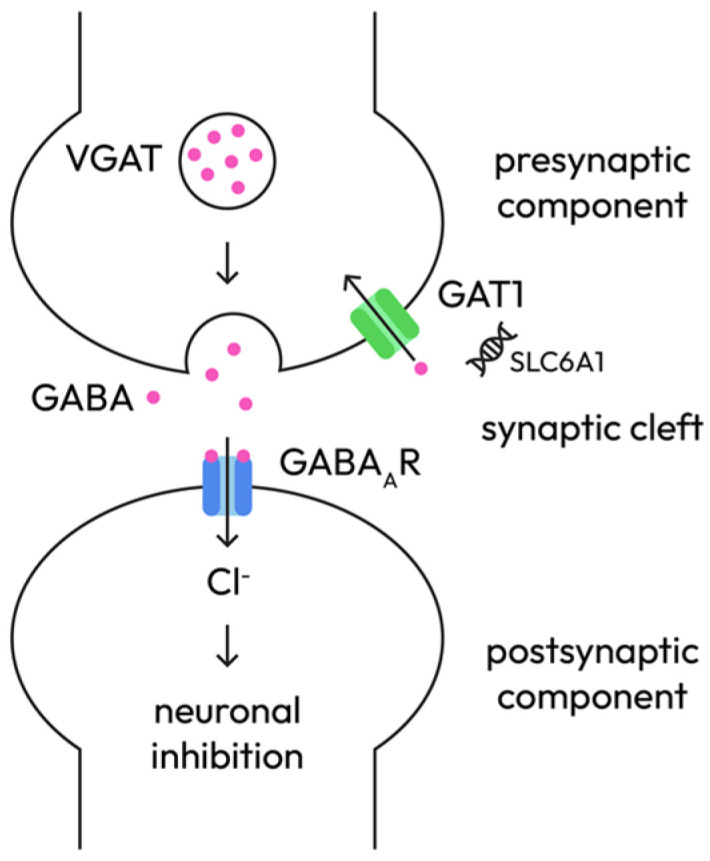
GABAergic Synapse. GABA = gamma-aminobutyric acid; GABA_A_R = GABA type A receptor; GAT1 = GABA transporter type 1; VGAT = vesicular GABA transporter. Activation of GABAergic neurons triggers the release of GABA from specialized vesicles into the synaptic cleft. Once released, GABA binds to its receptors on the postsynaptic component, triggering the influx of Cl^−^ ions, which results in neuronal inhibition. GABA reuptake occurs mainly via GABA transporters (GATs), such as GAT1, encoded by the SLC6A1 gene.

GABA is the main inhibitory neurotransmitter. GABAergic dysfunction reduces inhibitory tone and fosters hyperexcitability through presynaptic and postsynaptic mechanisms (Figure 3), thereby underpinning syndromes such as GEFS+ and absence epilepsy.

Presynaptic mechanisms:

- Interneuron dysfunction: SCN1A loss-of-function in Dravet syndrome preferentially affects GABAergic interneurons, leading to reduced inhibitory output and network disinhibition [29].
- Impaired GABA synthesis: reduced GAD65/67 expression decreases GABA availability [30].
- Vesicular transport deficiency: SLC32A1 (VGAT) mutations impair vesicular GABA packaging, leading to decreased synaptic inhibition [31].
- Synaptic vesicle cycling: SYN1 and STXBP1 mutations [32] affect vesicle docking and release.

Postsynaptic mechanisms:

- GABRA1, GABRB3, GABRG2, and GABRD mutations reduce inhibitory efficacy and are associated with generalized epilepsies and epilepsy syndromes (e.g., childhood absence epilepsy, GEFS+) [33].
- Low intracellular chloride determines the shift from inhibition to excitation: KCC2 (encoding for chloride exporter) downregulation or NKCC1 (encoding for chloride importer) upregulation causes depolarizing GABA responses [33].
- Chronic hyperexcitability induces compensatory downregulation of inhibitory synapses, weakening inhibitory tone further; activity-dependent remodeling alters benzodiazepine response [34].

Therapeutic implications of the novel insights regarding the GABAergic signaling pathway (Table 3) include the emerging insight that targeting chloride homeostasis (KCC2 enhancers) may restore the inhibitory action of GABA, especially in refractory epilepsy and early-life seizures.

**D.**  Additional pathways

GPCR and cAMP signaling: GNAO1, GNB1, and ADCY5 mutations cause epileptic encephalopathies and movement disorders [39].

Wnt/β-Catenin signaling pathway: This pathway orchestrates neurodevelopment and synaptic formation; mutations in CTNNB1 and related genes are linked to cortical malformations.

Ubiquitin-Proteasome System: This system controls protein degradation and turnover; disruption leads to accumulation of misfolded proteins and neuronal dysfunction. UBE3A mutations cause Angelman syndrome, in which loss of function leads to severe DEE (developmental and epileptic encephalopathies).

MAPK/JNK signaling and neuronal senescence: The JNK pathway regulates apoptosis, neuroinflammation, and synaptic remodeling. Dysregulation of this pathway can trigger senescence-like phenotypes in neurons, exacerbating network dysfunction in epilepsy [40]. Neuronal senescence is characterized by permanent exit from the cell cycle despite neurons being post-mitotic, increased expression of senescence markers such as p16INK4a, p21CIP1/WAF1, accumulation of DNA damage and oxidative stress, SASP (the senescence-associated secretory phenotype), including pro-inflammatory cytokines (IL-6, IL-1β), chemokines, and proteases, and altered synaptic function and neuronal metabolism [41]. Recurrent seizures activate JNK in hippocampal/cortical neurons, promoting transcription of pro-apoptotic and senescence-related genes [42].

Therapeutic strategies of MAPK/JNK-mediated neuronal senescence are depicted in Table 4. JNK pathway inhibitors reduce seizure frequency and neuronal damage in animal models, while senolytic drugs represent a novel strategy to clear senescent cells and ameliorate epileptic pathology.

### 3.4. Epigenetic Mechanisms in Epilepsy

Large-scale shifts in gene expression in epilepsy are driven by DNA methylation, histone modifications, and non-coding RNAs, which reshape neuronal structure, ion channel abundance, synaptic plasticity, and inflammatory signaling [46]. DNA hypermethylation and histone acetylation changes emerge after epileptogenic insults and persist in chronic epilepsy, stabilizing aberrant networks [47]. Epigenetic genes (writers/readers/erasers of chromatin marks, chromatin remodelers) themselves carry pathogenic variants in many epilepsies, directly tying the genetic architecture to epigenetic control.

A. DNA methylation

DNA methylation occurs primarily at CpG dinucleotides. The effects differ depending on the methylation site: promoter methylation generally silences transcription, whereas gene body methylation can promote transcription from alternative promoters or affect splicing. Methylation persistence is context-specific, ranging from transient to lifelong.

Non-CpG methylation is much less studied. CpH methylation (H = A, T, C) is prevalent in mature neurons, suggesting unique brain-specific regulatory roles. Although CpG methylation provides basal repressive marks, the biological impact of non-CpG methylation remains under investigation [47].

Methyl-CpG-binding protein 2 (MeCP2) is preferentially expressed in the brain and reads both 5-mC and 5-hmC marks. MeCP2 mutations cause Rett syndrome, but also interact with long non-coding RNAs, facilitating target recognition [47].

DNA methylation changes in epilepsy. The epigenetic status was investigated in Human Temporal Lobe Epilepsy (TLE) and revealed clear modifications: increased DNMT1 and DNMT3A expression is observed in the temporal neocortex and hippocampus; enhanced DNA methylation levels correlate with disease progression; 146 genes show altered methylation in TLE with hippocampal sclerosis; and most changes represent hypermethylation [46].

Gene-specific alterations in methylation were observed for RELN (increased promoter methylation associates with granule cell dispersion, a common migration defect in TLE), CPA6 (increased promoter methylation in TLE patients with a history of febrile seizures), BDNF (decreased methylation following status epilepticus, correlating with increased expression), GRIN2B (increased methylation reduces NMDA receptor subunit expression), and GluA2/GRIA2 (altered methylation affects AMPA receptor expression) [48].

GWASs (Genome-Wide Studies) on animal models using methyl-seq and array-based analyses revealed a specific DNA methylation signature that distinguishes chronic epileptic animals from controls: hypomethylation of 275 genes and hypermethylation of 13 genes in the KA mouse model; and hypomethylation of 1121 loci and hypermethylation of 1452 loci in chronic pilocarpine rat epilepsy [47].

DNA methylation is evaluated as an epigenetic biomarker for clinical use. Aberrant DNA methylation patterns consistently observed in epileptic brain tissue are being explored for diagnosis, prognosis, and stratification of pharmacoresistance [48]. Matched brain–blood methylation profiling in focal cortical dysplasia shows shared signatures as specific CpG biomarkers that distinguish FCD subtypes with high accuracy, supporting peripheral epigenetic diagnostics [49] (e.g., protein CHORDC1 in peripheral blood was identified as a potential biomarker).

DNA methylation changes crosstalk with genetic pathways. MeCP2 (methyl CpG-binding protein 2) binds methylated DNA and recruits HDACs (histone deacetylases), HMTs (histone methyltransferases), and HDMTs (histone demethylases), linking DNA methylation to histone modifications. Mutations disrupt this integration, leading to aberrant expression of multiple target genes (e.g., contributing to RELN hypermethylation in TLE). DNMT variants alter SAM availability, and hyperhomocysteinemia-induced hypomethylation exacerbates neuronal damage [47].

B. Histone modifications

Histones are basic proteins forming nucleosome cores around which DNA wraps. The N-terminal tails contain multiple sites for posttranslational modifications (PTMs): acetylation (neutralizes positive charge, relaxing chromatin structure and is generally associated with transcriptional activation), methylation (can activate or repress depending on residue and methylation state: H3K27me3/H3K9me3 are repressive marks while H3K4me/H4K12ac are activating marks), phosphorylation (rapid and dynamic in response to neuronal activity, usually associated with transcriptional activity), ubiquitination and other post-translational modifications. The combination of these marks forms a “histone code” influencing transcriptional state [47].

Histone modification changes in epilepsy. Following seizures, there are acute changes: a rapid increase in H3 phosphorylation (H3-S10) occurs in dentate granule neurons after KA (kainic acid) seizures, while H4 acetylation appears later, spreading throughout hippocampal subfields; modifications associate with immediate early gene (IEG) induction (c-fos, c-jun); or SIRT1 activation initiates H4K16ac depletion following status epilepticus.

In chronic epilepsy, different changes have been described: upregulated HDAC2 expression in experimental models and in human TLE; increased expression of HDAC1, 2, 3, and 8; or increased expression of HDAC5 in hippocampal granule cells [46].

Histone modifications crosstalk with genetic pathways. Histone modifications were linked to epilepsy in different ways: histone acetylation/methylation altered patterns of regulation of immediate early genes (IEGs) involved in synaptic plasticity; increased histone H3 lysine 9 acetylation (H3K9ac) correlates with seizure-induced gene activation [50]; and histone deacetylase (HDAC) inhibitors exhibit anticonvulsant effects in animal models. New insights reveal that histone modifications are associated with activity-dependent gene expression: seizure activity induces dynamic chromatin remodeling [51], facilitating rapid transcriptional responses and the activity-dependent recruitment of chromatin remodelers to IEG promoters, which modulates plasticity but may also reinforce hyperexcitability.

Decreased H4 acetylation at the GluA2 locus reduces gene expression. Increased H4 acetylation at BDNF promoter follows status epilepticus. Altered acetylation and methylation patterns regulate synaptic plasticity genes. Some of the altered histone modification patterns are depicted in Table 5.

**Genetic epilepsy syndromes with histone modifications.**

Several epilepsy syndromes result from mutations in genes encoding histone-modifying enzymes. Detailed description of these syndromes is beyond the scope of this paper, but we will shortly present some as examples [46]:

- Kleefstra syndrome: Mutations in EHMT1 (euchromatin histone methyltransferase 1) target H3K9me and H3K9me2, promoting gene repression. Haploinsufficiency or duplication causes decreased global methylation and associated clinical features like intellectual disability, seizures, and behavioral abnormalities.
- Kabuki syndrome: Mutations in KMT2D (H3K4-specific methyltransferase) alter gene regulation and associate with a neurodevelopmental disorder with epilepsy.
- Nicolaides–Baraitser Syndrome: Mutations in SMARCA2 (BAF remodeling complex) associate neurodevelopmental disorder with intellectual disability and seizures.
- ATRX Syndrome: ATRX is a SWI/SNF family member containing a DNMT3-homologous domain and interacts with MeCP2, EZH2, and Daxx. Mutations cause X-linked intellectual disability with seizures.

**Histone modification as a therapeutic target.**

Preclinical and clinical studies [52] evaluated the use of HDAC inhibitors, with encouraging results, as follows:

- Valproic acid: exhibits a dual mechanism (HDAC inhibition + effects on GABA ion channels);
- Trichostatin A: prevents GluA2 deacetylation but lacks anticonvulsant effects;
- SAHA (suberoylanilide hydroxamic acid): exhibits neuroprotective effects and reduces aberrant neurogenesis;
- Sodium butyrate: increases H3/H4 acetylation in hippocampus and cortex;
- Curcumin: blocks acetylation and c-fos induction.

Currently, there are limitations to using these agents as daily medication: HDAC inhibitors lack specificity, targeting enzymes globally rather than specific modifications, leading to off-target effects.

Future directions should address these limitations and could focus on developing isoform-selective HDAC inhibitors, targeting specific histone readers or writers, or on standard combination therapies with traditional antiseizure medications.

C. Chromatin remodeling enzymes

Chromatin remodeling influences DNA accessibility and gene transcription via ATP-dependent remodeling complexes and histone modifications. Mutations or altered expression of these factors can disrupt chromatin states and transcriptional homeostasis, contributing to epileptogenesis.

ATP-dependent chromatin remodeler families include SWI/SNF, ISWI, CHD, and INO80, which reposition nucleosomes to regulate transcription [53]. These remodeling enzymes use ATP hydrolysis to disrupt histone-DNA contacts, restructuring nucleosomes and regulating DNA accessibility [54].

The SWI/SNF Family includes subunits relevant for epileptogenesis, such as the BAF complex (SMARCA2 mutations cause Nicolaides–Baraitser syndrome), ARID1B, which is frequently mutated in neurodevelopmental disorders with epilepsy [55], and ATRX (DNMT3-homologous domain).

CHD Family subunits are associated with active transcription and H3.3 variant deposition and include CHD2 (disruption leads to photosensitive epilepsy via impaired histone remodeling and DNA repair [54]), CHD6 (described in connection with Pitt–Hopkins syndrome), and CHD7 (described in connection with CHARGE syndrome).

ISWI and INO80 Families are less well-studied in epilepsy but critical for chromatin organization.

**Chromatin remodeling crosstalks with genetic and epigenetic pathways.**

ATRX demonstrates convergence among chromatin remodeling, DNA methylation, and histone modification pathways and associates with repressive chromatin, MeCP2 (a DNA methylation reader), EZH2 (a histone methyltransferase), and Daxx (a death-associated protein).

Activity-dependent remodeling may reinforce hyperexcitability through distinct mechanisms: seizure activity induces dynamic chromatin remodeling, remodeling processes facilitate rapid transcriptional responses, and the recruitment of remodelers to IEG promoters may be activity-dependent.

Histone modifications interact with lncRNAs (long non-coding RNAs), creating functional links between chromatin-remodeling complexes and lncRNAs that influence gene regulation [46].

D. Non-coding RNA regulation

**MicroRNA networks.**

MicroRNAs (miRNAs) are 19–24-nucleotide regulatory RNAs that mediate posttranscriptional gene silencing. Over 100 miRNAs are altered in the epileptic hippocampus. miRNAs exert diverse effects, including on neurogenesis and differentiation, as miR-124 and miR-137 (co-administration prevents hippocampal neural stem cell loss during non-convulsive seizures, and overexpression restores NSC changes); on synaptic plasticity, as miR-129-5p (silencing prevents synaptic shrinkage and reduces seizure severity); on apoptosis regulation, as miR-136 (overexpression reduces seizure frequency and duration, improves hippocampal damage, reduces apoptosis and inflammation) or miR-184 (modulates seizure-induced neuronal death); and on inflammatory response, as miR-146a (silencing reduces IL-1β, IL-6, and IL-18 expression, alleviates oxidative stress and inflammatory response, and protects against neuronal damage) [48].

MicroRNAs were studied for their therapeutic potential. Miravirsen is the first oligonucleotide targeting a miRNA to be tested in clinical trials. AntagomiR-134 showed effectiveness in preclinical models, reducing SE duration and preventing spontaneous recurrent seizures. miR-134 is abnormally expressed in the plasma and CSF of epilepsy patients, suggesting biomarker potential and possible clinical translation [48].

**Long non-coding RNA networks.**

Long non-coding RNAs (lncRNAs) exceed 200 nucleotides and predominantly localize to nucleus, consistent with epigenetic regulatory roles. Over 100,000 lncRNAs were identified with unique expression patterns in hippocampus. An extensive presentation of the lncRNAs exceeds the scope of this paper, so we will shortly present some representatives that showed connections with epileptogenesis. NEAT1 (Nuclear Enriched Abundant Transcript 1) is highly expressed in neurons; it is significantly elevated in epileptic cerebral cortex and briefly reduced during the acute phase of pilocarpine-induced epilepsy. Evf2 regulates transcription factors crucial for GABA-producing interneuron development; knockout mice exhibit increased seizure susceptibility, impaired GABAergic neural circuits, and more severe, frequent seizures. PVT1 (Plasmacytoma Variant Translocation 1) is highly expressed in hippocampal tissue of epileptic rats; silencing PVT1 suppresses astrocyte activation and downregulates Wnt signaling. Natural Antisense Transcripts (NATs), a subset of lncRNAs, originate from the opposite strand of sense RNA and usually block the expression of coding gene by recruiting epigenetic silencing machinery; oligonucleotides can target and modulate NATs, can increase SCN1A mRNA and prevent seizures in Dravet mouse models, and entered clinical trials [47].

**Circular RNAs**

Circular RNAs (circRNAs) are covalently closed, highly stable molecules significantly enriched in the nervous system. They act as miRNA sponges, serve as molecular scaffolds for RNA-binding proteins, control transcription of host genes, and can directly synthesize short peptides/proteins. Epilepsy-related circRNAs were described, including 100 differentially expressed circRNAs in the epileptic mouse hippocampus or 586 differentially expressed circRNAs in the TLE patient brain tissue. CircRNA-0067835 is significantly abnormally expressed in epileptic tissue and plasma, and lower expression is associated with higher seizure frequency. Circ_0001293/miR-8114/TGF-β2 axis significantly prevents epilepsy progression. Structural stability and abundant expression suggest that circRNAs are effective biomarkers for epilepsy and neurological diseases, though this has not yet been established [48].

E. NRSF/REST—Master Epigenetic Regulator

NRSF (neuron-restrictive silencing factor)/REST (RE-1 silencing transcription factor) is described as a “master regulator” that mediates widespread alterations in gene expression in epilepsy. It is a transcriptional repressor that alters the chromatin landscape by recruiting HDACs, HMTs, and HDMTs. Primarily represses neuronal-specific genes in non-neuronal tissue, target genes silenced through binding to NRSE/RE1 region, and recruits CoREST and mSin3A plus other factors. Epilepsy shows different activity: it is upregulated in the CA3 hippocampal region following kainic acid-induced SE, regulates HCN1 channel (HCN1 persistently down-regulated following seizures and contributes to epilepsy development), and contributes to epilepsy by repressing KCC2 (potassium-chloride cotransporter) and GRIN2A (NMDA receptor subunit) [46].

F. RNA modifications

RNA methylation is a post-transcriptional modification that affects mRNA stability, translation, splicing, and nuclear export. 5-Methylcytosine (5-mC) contributes to RNA stability and mRNA translation. N6-Methyladenosine (m6A) is closely associated with nervous system development and can regulate non-coding RNAs implicated in epilepsy. RNA modifications can alter ncRNA function in epilepsy, affect translation of ion channel and receptor mRNAs, modulate inflammatory signaling molecules and influence synaptic protein synthesis. Research remains nascent but promises new mechanistic insights [48].

G. Zinc finger transcription factors (ZNF)

ZNF domains enable sequence-specific DNA binding; ZNF proteins modulate neuronal differentiation, synaptic development, and neuroinflammation, influencing epileptogenesis. ZEB2, a transcriptional repressor with zinc fingers, is implicated in neurodevelopmental delay and seizure susceptibility [53]. Recent studies identify mutations in ZNF142 [54], ZNF711 [55], and other ZNFs associated with early-onset epileptic encephalopathies. Mutations in KDM5C, a histone demethylase interacting with zinc finger factors, alter chromatin accessibility and increase seizure propensity [56].

The therapeutic implications of the epigenetic mechanisms underlying epilepsy are summarized in Table 6. Epigenetic therapies targeting chromatin state and ZNF factors hold promise for disease modification in refractory epilepsy, complementing traditional anti-seizure drugs.

### 3.5. Gene-Environment Interactions Mediated by Epigenetics

Environmental factors interact with genetic predisposition through epigenetic mechanisms, thereby dynamically modifying disease risk. When environmental triggers occur, the epigenetic response [48].

Early-life seizures, like febrile seizures and early status epilepticus, induce lasting DNA methylation changes. Histone modification alterations persist long after the initial insult and may determine progression to chronic epilepsy. Neuroinflammation from infections or autoimmune processes alters DNA methylation at inflammatory gene promoters, histone acetylation at cytokine genes, and the expression of inflammation-regulatory miRNAs.

Metabolic factors showed effects on epileptogenesis. A ketogenic diet alters global DNA methylation via adenosine signaling and induces metabolic changes that affect SAM availability. Genome-wide methylation profiling shows decreased methylation after 4 and 12 weeks of ketogenic diet, with genes related to epilepsy and inositol metabolism affected. Hyperhomocysteinemia affects SAM availability, reduces methylation capacity, and exacerbates neuronal damage. Nutrients influence DNMT activity, while valproic acid inhibits DNMT, leading to decreased overall DNA methylation [48].

The latent period between initial insult (status epilepticus, trauma, febrile seizures) and recurrent seizures represents a critical therapeutic window. Maximal DNA methylation changes occur during the latent period, as do histone modifications (most dynamic before epilepsy onset), changes in non-coding RNA expression (dramatic shifts), and progressive DNA methylation, which correlate with disease duration. This period proved the optimal therapeutic window for DNMT inhibition to prevent pathological methylation, HDAC inhibitor treatment to maintain beneficial gene expression, antagomiR delivery to block pro-epileptogenic miRNAs and adenosine augmentation to reverse hypermethylation [48].

### 3.6. Genetic Causes of Epilepsies and Actionable Genes

#### 3.6.1. Genetic Causes

Genetic causes of epilepsies are a crucial area of research in understanding the underlying mechanisms of this neurological disorder. Epilepsies can arise from mutations in various genes that play critical roles in brain development, neuronal signaling, and ion channel functions. These genetic mutations can disrupt normal brain function in several ways.

For example, genetic mutations influencing brain development can result in structural anomalies that potentially predispose to seizure susceptibility. These anomalies may manifest as alterations in neuronal connectivity or cortical developmental malformations, both of which can increase the propensity for epilepsy onset.

To maintain the balance between excitation and inhibition in the brain, genes that regulate neuronal signaling are essential. Mutations in these genes can cause an imbalance, leading to excessive neuronal firing and, consequently, seizures. For example, genes that encode neurotransmitter receptors or enzymes involved in neurotransmitter metabolism are critical in this regard.

Genetic epilepsies often involve ion channel genes, crucial for controlling ion movement across neuronal membranes. This process is vital for initiating and spreading electrical signals in the brain. Mutations in these genes can impair ion channel function, leading to abnormal neuronal electrical activity and increased susceptibility to seizures. Notably, mutations affecting sodium, potassium, and calcium channels are particularly significant because they directly generate action potentials and transmit signals between nerve cells.

Overall, the genetic basis of epilepsies highlights the complex interplay of various biological pathways that maintain normal brain function. Understanding these genetic factors not only aids in the diagnosis and classification of different types of epilepsy but also opens potential avenues for targeted therapies and personalized treatment approaches.

From a genetic and phenotypic perspective, epilepsies are highly diverse, with pathogenic variants including nonsense, missense, splice-site mutations, and small intragenic deletions or insertions [59].

The concept of electroclinical syndromes captures the clinical patterns of most generalized epilepsies. The electroclinical syndromes of childhood absence epilepsy, juvenile myoclonic epilepsy, and juvenile absence epilepsy are among the few neurodevelopmental disorders that show clear evidence of genetic etiologies. Twin studies have shown concordance of over 90% in identical twins, which is much higher than in dizygotic twins, indicating that most causative factors are genetic at the population level [60].

#### 3.6.2. Inheritance Patterns

The genetic basis of epilepsy is a multifaceted area of study that involves understanding how genetic mutations and hereditary factors contribute to the development of this neurological disorder.

While some forms of epilepsy can be attributed to environmental factors or acquired conditions, a significant proportion is linked to genetic causes, as monogenic (single gene) or polygenic (multiple genes) etiology. Genetic causes of the epilepsies include a variety of inheritance patterns and specific disorders (Table 7).

Mendelian inheritance patterns are observed in several forms of epilepsy. Autosomal dominant epilepsies, such as benign familial neonatal epilepsy (BFNE) and autosomal dominant nocturnal frontal lobe epilepsy (ADNFLE), exhibit a 50% inheritance risk for first-degree relatives, with a high clinical manifestation rate (approximately 85% for BFNE and 70% for ADNFLE) [61]. Autosomal recessive epilepsies, including Lafora disease and Unverricht-Lundborg disease, are severe and carry a 25% inheritance risk for siblings, with higher prioritization in cases of parental consanguinity [62].

X-linked epilepsies also show distinct inheritance patterns. X-linked recessive disorders, such as ARX-associated epilepsies, predominantly affect boys [63]. X-linked dominant disorders, including double cortex syndrome and periventricular nodular heterotopia, affect girls, whereas boys are often severely affected or may not survive embryonic development. Additionally, girl-specific disorders, such as PCDH-19-linked epilepsy, result in healthy male carriers and affected female carriers [64].

Non-Mendelian inheritance: Most cases of epilepsy follow non-Mendelian inheritance, suggesting lower risks for relatives. However, the exact genetic architecture of these disorders remains uncertain, including the number of genes involved and the magnitude of their effects. In common epilepsies, empirical risks are well documented. For GGE, first-degree relatives have an estimated risk 8 times that of the general population, while second-degree relatives have a considerably lower risk. For focal epilepsies, first-degree relatives face a risk approximately 2.5 times higher than the general population [61].

Notable exceptions to these rules exist in which a mutant gene is inherited in a Mendelian fashion and causes common epilepsy phenotypes. For instance, about 1% of individuals diagnosed with GGE have mutations in the SLC2A1 gene, which encodes the glucose transporter GLUT1 [65]. In focal epilepsies, the most common mutations occur in the LGI1 and DEPDC5 genes. These mutations are responsible for familial focal epilepsy with variable foci (FFEVF) and familial lateral temporal lobe epilepsy, respectively [66].

Monogenic epilepsy syndromes are typically inherited in a Mendelian fashion, meaning they follow specific patterns of inheritance such as autosomal dominant, autosomal recessive, or X-linked. These syndromes are often caused by mutations in a single gene, and the resultant phenotype can be traced directly to these genetic alterations. The most important monogenic epilepsies are depicted in Table 8.

Polygenic epilepsy: In contrast, polygenic epilepsy arises from the interaction of numerous genetic variations, each contributing incremental risk to epilepsy development. This type of epilepsy does not adhere to Mendelian inheritance patterns and results from the combined effects of multiple genes, each making a modest contribution. Progress in genome-wide association studies (GWAS) has unveiled numerous genetic regions linked to epilepsy, revealing the intricate genetic landscape of these disorders. Polygenic forms of epilepsy typically exhibit diverse clinical manifestations influenced by both genetic predispositions and environmental factors [79].

#### 3.6.3. Specific “Actionable” Genetic Findings

Dravet syndrome, caused by mutations in the SCN1A gene in 80% of cases, can be diagnosed relatively easily after the first few years of life, when characteristic seizure patterns and developmental stasis emerge. Early testing should be considered, as evidence suggests that early, aggressive treatment may improve prognosis. Early diagnosis can enhance seizure management and improve cognitive outcomes. Effective treatments include valproic acid, topiramate, stiripentol, and clobazam, which have demonstrated efficacy. It is advisable to avoid sodium channel blockers such as lamotrigine and carbamazepine. These recommendations are largely informed by findings from case series, except for stiripentol, for which controlled trials have substantiated its beneficial effects [80].

Epileptic encephalopathies linked to mutations in the SCN2A and SCN8A genes are now better defined and occur in a smaller proportion than Dravet syndrome. Their drug response profiles may vary, as the encoded proteins localize to excitatory neurons, whereas the SCN1A protein appears to be present in inhibitory interneurons. In SCN8A encephalopathy, sodium channel blockers have been effective in some cases [81].

Mutations in the SLC2A1 gene that cause severe infantile encephalopathy with hypoglycemia may partially respond to the ketogenic diet. Many patients with mild GLUT1 deficiency respond to conventional antiepileptic drugs, but for refractory cases, ketogenic diet therapy should be emphasized.

The novel medication ezogabine (retigabine) is designed to address deficiencies in the KCNQ2 potassium channel gene, which are associated with both benign familial neonatal epilepsy (BFNE) and severe epileptic encephalopathy due to spontaneous mutations. However, its clinical utility may be limited by toxicities affecting the skin, nails, and retina. Sodium channel blockers such as carbamazepine and phenytoin have also shown efficacy in managing this condition.

Pyridoxine-dependent epilepsy is a rare form of childhood epilepsy that is generally diagnosed by clinical suspicion and response to pyridoxine. Confirmation can be provided by detecting mutations in the ALDH7A1 gene and can also be used to screen at-risk relatives. Disorders associated with pyridoxamine 5′-phosphate oxidase (PNPO) deficiency may respond to treatment with pyridoxal 5′-phosphate alone; however, the relationship between specific PNPO mutations and treatment response remains uncertain [82].

A clinical response to quinidine has been observed in epilepsy with focal migratory seizures in infants. Quinidine reverses the increase in in vitro function observed in mutations in the KCNT1 gene. Similarly, memantine therapy appeared effective in patients with a mutation in the GRIN2A gene, which encodes a subunit of the glutamate receptor. The recent discovery of the importance of mutations in the DEPDC5 gene, now known to regulate mTOR, suggests the possibility of treatment with rapamycin analogs. However, to establish efficacy, all these observations will require rigorous double-blind studies [6].

Recent innovations in genetic testing technologies, such as next-generation sequencing (NGS), have significantly eased the discovery of actionable genes in clinical practice. These advancements empower clinicians to diagnose precise genetic epileptic syndromes, forecast disease trajectory, and customize treatment plans accordingly. Personalized medicine initiatives aim to improve therapeutic outcomes by addressing the underlying genetic origins, potentially improving seizure management and overall patient well-being.

In conclusion, the identification of specific actionable genes in genetic epileptic syndromes has transformed our understanding of these disorders and opened avenues for targeted therapies. Continued research into the molecular mechanisms underlying these syndromes promises to uncover new therapeutic targets and improve outcomes for individuals affected by genetic epilepsy.

### 3.7. Genetic-Epigenetic Crosstalk in Specific Epilepsy Syndromes

#### 3.7.1. Dravet Syndrome

The genetic basis of Dravet syndrome is SCN1A mutations in 80% of cases [80], most of which are de novo and cause truncated proteins. Clinical features include a typical onset within the first year of life, with initial symptoms as febrile seizures progressing to severe epilepsy. The seizure types vary from myoclonic and tonic–clonic to absence. Patients associate developmental delays, cognitive challenges, and behavioral complexities. Dravet syndrome is typically resistant to conventional antiepileptic treatments.

SCN1A expression is modulated by histone modifications and, together with DNA methylation patterns that influence phenotypic variability, constitutes the epigenetic particularities of Dravet syndrome.

Treatment needs require a multifaceted approach. In addition to well-established effective medications (valproic acid, topiramate, stiripentol, clobazam), there are medications to avoid (sodium channel blockers—lamotrigine, carbamazepine) and approved novel add-on therapies (cannabidiol and fenfluramine). The ketogenic diet has demonstrated effective outcomes. Emerging therapies (e.g., CRISPR/Cas9 and ASO therapies—zorevunersen/STK-001) show promise in clinical trials. In general, early diagnosis is crucial for seizure management and cognitive outcomes, and early, aggressive treatment may improve prognosis [83].

#### 3.7.2. Lennox–Gastaut Syndrome (LGS)

It has an intricate genetic and epigenetic basis with diverse origins, including genetic mutations, structural brain anomalies, and perinatal complications. Genetic mutations in GABRB3 and ALG13 were detected, while epigenetic factors modulate severity. Onset is usually at 3–5 years of age, with various seizure types (tonic, atonic, atypical absence) and cognitive deficits. A distinct EEG signature with slow spike-wave discharges helps diagnose and differentiate LGS from other entities.

Treatment is rather complex, often requiring a combination of antiepileptic drugs (valproate, lamotrigine, levetiracetam), dietary therapies (ketogenic diet), and surgical interventions (corpus callosotomy, vagus nerve stimulation) [84].

#### 3.7.3. Childhood Absence Epilepsy (CAE)

CAE typically manifests at 4–10 years of age as frequent absence seizures and brief episodes (a few seconds) of staring and unresponsiveness. Genetic etiology consists of mutations in genes involved in neuronal signaling (CACNA1H, GABRG2). Epigenetic modulation consists of histone modifications at GABA receptor genes and responsiveness to DNA methylation changes.

Ethosuximide, valproate, lamotrigine are included in the standard treatment protocols. Prognosis is generally good, many children outgrow seizures by adolescence [85].

#### 3.7.4. West Syndrome

Genetic and epigenetic factors are associated with mutations in ARX and STXBP1, structural brain abnormalities, metabolic disorders, and perinatal insults. The syndrome is characterized by clusters of spasms in the first year of life, accompanied by hypsarrhythmia on EEG. Treatment protocol includes ACTH (adrenocorticotropic hormone), corticosteroids, and Vigabatrin. Prognosis depends on the underlying etiology [86].

### 3.8. Diagnostic and Genetic Testing

The diagnosis of genetic epilepsy syndromes involves a comprehensive approach that includes clinical evaluation, electroencephalography (EEG), neuroimaging, and genetic testing. A detailed medical history, including family history, is crucial in identifying potential hereditary patterns and guiding the diagnostic process [87].

- Clinical evaluation and EEG

The clinical assessment centers on the patient’s seizure chronicle, developmental progress, and any related neurological or cognitive deficits. EEG plays a crucial role in epilepsy diagnosis by identifying distinct patterns of brain electrical activity associated with particular epilepsy syndromes. For instance, the presence of slow spike-wave discharges in Lennox–Gastaut Syndrome or the characteristic 3 Hz spike-and-wave patterns observed in Childhood Absence Epilepsy illustrate this diagnostic capability [87].

- Neuroimaging

Advanced neuroimaging methods, such as magnetic resonance imaging (MRI), are used to detect structural abnormalities in the brain that may be implicated in epilepsy. Although genetic epilepsy syndromes frequently exhibit normal MRI results, identifying structural lesions can aid in distinguishing genetic epilepsy from other types of the condition [87].

- Genetic testing

Genetic testing is now integral in diagnosing genetic epilepsy syndromes. Technologies such as whole-exome sequencing (WES) and targeted gene panels are frequently used to identify harmful mutations. WES scrutinizes mutations across the entire coding regions of the genome, whereas targeted gene panels focus on a defined set of genes linked to epilepsy. Identifying a genetic mutation can confirm a diagnosis, provide prognostic insights, and guide treatment strategies. Genetic counseling is advised for families affected by discussing the ramifications of genetic discoveries, inheritance probabilities, and reproductive choices [87].

### 3.9. Treatment and Management

The treatment and management of genetic epilepsy syndromes are tailored to the specific syndrome and the individual patient’s needs. While many antiepileptic drugs (AEDs) are available, their efficacy can vary significantly depending on the underlying genetic mutation [87].

#### 3.9.1. Pharmacological Treatment

Standard antiepileptic drugs are often the first line of treatment. For instance, ethosuximide is particularly effective for absence seizures in Childhood Absence Epilepsy, while valproate is commonly used for various types of seizures in Lennox–Gastaut Syndrome. However, certain medications may exacerbate seizures in specific syndromes. For example, sodium channel blockers like carbamazepine can worsen seizures in patients with Dravet Syndrome. Therefore, genetic testing can be instrumental in selecting the appropriate medication [87].

#### 3.9.2. Non-Pharmacological Therapies

Non-pharmacological interventions are indispensable, particularly for drug-resistant forms of epilepsy. The ketogenic diet, known for its high-fat, low-carbohydrate composition, has demonstrated significant efficacy in reducing seizure frequency across various genetic epilepsy syndromes, notably Dravet Syndrome and Lennox–Gastaut Syndrome. Vagus nerve stimulation (VNS) involves surgically implanting a device to activate the vagus nerve, offering a promising avenue for seizure control. Surgical options such as corpus callosotomy or resective surgery may be considered for patients presenting with focal lesions or experiencing refractory epilepsy that does not respond to conventional treatments [87].

#### 3.9.3. Emerging Therapies—CRISPR/CAS

New and innovative treatments, such as gene therapy and precision medicine, show potential in addressing the precise genetic origins of epilepsy. Gene therapy seeks to replace or correct the defective gene underlying the disorder, whereas precision medicine strategies tailor therapies to each person’s unique genetic makeup. Although these treatments are currently undergoing experimental phases, they mark a substantial leap forward in the quest to potentially cure or substantially enhance the prognosis for individuals affected by genetic epilepsy syndromes [87].

A method that could revolutionize the treatment of monogenic epilepsies in the future is genome editing with curative intent, using the CRISPR/CAS system. CRISPR/Cas9 is a natural bacterial immune system repurposed for mammalian genome editing, breaking double-stranded DNA with base-pair precision. Studies have shown promising results for CRISPR technology. For instance, upregulation of wild-type SCN1A allele expression resulted in a ~50% improvement in the epileptic phenotype [88]. But CRISPR can act in multiple ways.

**CRISPR/Cas9 mechanisms**

The most common uses of CRISPR/Cas9 technology in gene editing are:

- NHEJ (Non-homologous end joining): fast but error-prone, produces indels;
- HDR (Homology-directed repair): precise but less efficient;
- Base editing: nucleotide-level changes without double-strand breaks;
- Prime editing: insertions/deletions without DNA breaks or templates;
- Gene modulation: control of genome function without gene editing.

**Figure 4:**
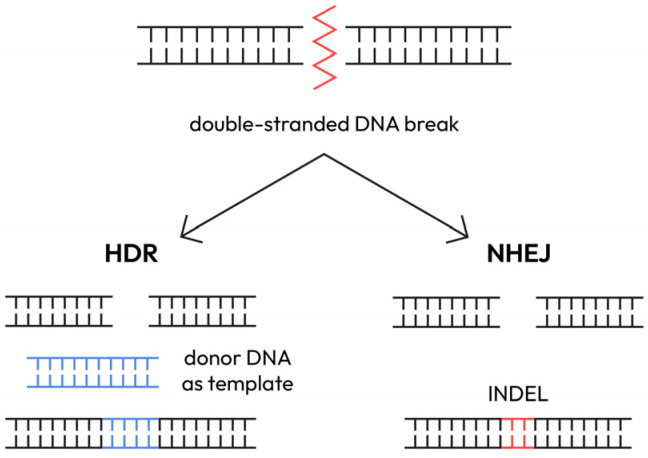
The CRISPR/CAS system. HDR = homology-directed repair; NHEJ = non-homologous end joining; INDEL = (small) insertion or deletion. Homology-directed repair (HDR) is a precise DNA repair mechanism that fixes double-strand breaks by using a donor DNA template through homologous recombination. Nonhomologous end joining (NHEJ) is a repair pathway that could result in small insertions or deletions (INDELs) at the break site.

The CRISPR/CAS system is depicted in Figure 4. CRISPR/CAS breaks double-stranded DNA with great precision at the loci of interest, with an accuracy of a base pair. In response, the cells activate the DNA repair mechanisms, either in a homologous repair way (HDR, homology-dependent repair) or a non-homologous repair way, by simply joining the two ends of the break (NHEJ, non-homologous end joining). The second method is the fastest repair option available to mammalian cells, including neurons; although it is more error-prone, it is preferred and most frequently used. The CRISPR/CAS technique exploits cells’ ability to repair DNA breaks, which are often followed by the formation of indels that selectively affect the targeted coding exon, introducing a frameshift or a nonsense mutation and altering the reading frame [88].

Generating double-stranded DNA breaks is not the only use of CRISPR/CAS. This system can be used to change DNA at a base pair level—“base editing”. Base editing can replace any nucleotide with another one, introducing a transition or a transversion mutation in the targeted DNA segment. For example, the base editing technique could be used to treat a dominant-negative mutation in the GABRG2 gene; in this case, other gene editing approaches would not be effective [88].

A newly described use of the CRISPR/CAS system is the “prime editing” method. This technique is extremely precise, allowing a wide variety of genetic modifications, from nucleotide conversions to the introduction of insertions or deletions, without breaking the double-stranded DNA or using a DNA template. Prime editing was associated with significantly reduced adverse effects, such as genotoxicity, compared with the classic approach [88].

CRISPR/CAS can modulate gene expression. Nuclease-dead Cas (dCas) molecules can be used to precisely control genome function without gene editing. Several modulator molecules bind to the dCas protein, which can bind to a specific locus. Thus, the regulatory molecules are guided to the target locus, where they exert specific functions in gene modulation by inhibiting or activating gene expression [89].

**Traditional CRISPR/Cas9 for genetic correction in epilepsy—application examples**

- SCN1A mutations (Dravet syndrome)

CRISPR/Cas9 was used for upregulation of wild-type SCN1A allele, followed by a 50% improvement in epileptic phenotype [88]. CRISPR/Cas9-based gene therapy targeting SCN1A in inhibitory neurons ameliorates epileptic and behavioral phenotypes in mouse models [18], increasing Nav1.1 expression in parvalbumin-positive GABAergic neurons. Upregulation of SCN1A expression in inhibitory neurons can improve symptoms, even when applied after the juvenile stage [18].

- iPSC-based disease models

CRISPR/CAS was used to study the mechanism of epilepsy caused by an SCN1A loss-of-function mutation in an Induced Pluripotent Stem Cell (iPSC)-based disease model, revealing how the mutation affects Nav1.1 channels in GABAergic neurons: the mutation reduces sodium current density and shifts the activation curves in GABAergic neurons from inhibitory to excitatory dominance. The CRISPR/CAS technology reveals the physiological basis of epilepsy caused by SCN1A mutations and provides guidance for clinical drug administration, cautioning against sodium channel-blocking antiepileptic drugs [90].

- Poison exon removal

Dravet syndrome can be caused by the “poison” exon 20N within the introns of the SCN1A gene, leading to reduced levels of full-length SCN1A protein. CRISPR/CAS technique could be directed towards the splicing site of this exon, to prevent a non-functional transcript [91].

- Deletion reversal:

The CRISPR/CAS system can be used to reverse a deletion-causing disease. The missing DNA sequence in the NRXN1 epilepsy gene could be reinserted in two ways: using the HITI (homology-independent targeted integration) or MMEJ (homology-mediated end-joining) techniques [92].

- KCNA1 upregulation

CRISPR/Cas9 can increase expression of the potassium channel gene in mouse hippocampal excitatory neurons, leading to substantial decreases in neuronal excitability, reduced seizures, rescued cognitive impairment, and rescued transcriptomic alterations in the temporal lobe epilepsy model. The study provides proof-of-principle for a CRISPR-based approach to treat neurological diseases with abnormal circuit excitability [93].

**Challenges:**

CRISPR/CAS is not yet a standardized procedure for the treatment of epilepsy. Studies are needed to elucidate the most suitable use of the technique in clinical practice, with maximum efficiency and minimal side effects. Despite advantages over conventional gene therapy, the CRISPR/CAS system showed some limitations [88]:

- Difficulties in delivering to the central nervous system (due to immunological and physical reasons)—requires surpassing the blood–brain barrier;
- Editing efficiency—low efficiency can lead to pathogenic mosaicism, which can prove pathogenic;
- Off-target effects registered in other locations besides the targeted sites;
- Immunogenicity concerns;
- The technique is most suitable for monogenic epilepsies.

Human clinical trials using CRISPR/CAS technology currently address CAR-T receptors (chimeric antigen receptors), hematological diseases and eye pathology [94]. Important aspects need to be further solved in order that CRISP/Cas therapies to become standard of care for human pathologies, including the most suitable viral vector type, with tissue specificity, diminish immunogenicity and enough cargo capacity. The most efficient vector system for CRISPR/Cas components today is a dual AAV9 viral vector (low immunogenicity, though cargo capacity limited). Other vectors studied are adenoviruses and lentiviruses [93].

**dCas9 for epigenetic modulation**

CRISPR can also intervene at the epigenome level. Nuclease-dead Cas9 (dCas9), fused to epigenetic effectors, enables site-specific modifications without DNA cleavage. For example, dCas9 associated with a demethylase (TET1) can direct the demethylase to the binding site, leading to local demethylation of the CDKL5 gene promoter and subsequent activation of the targeted gene [95]. Some of the epigenetic modulations are listed below:

- DNA methylation editing:

The dCas9-DNMT3A complex establishes methylation at specific loci. On the contrary, dCas9-TET1 complex removes methylation, reactivating silenced genes and activating targeted genes in laboratory conditions. Example: CDKL5 promoter demethylation in infantile epilepsy [46].

- Histone modification editing:

The dCas9-HAT complex increases local acetylation. On the contrary, dCas9-HDAC complex removes acetyl groups. dCas9-HMT/HDM complex controls methylation states [57].

- Transcriptional modulation:

dCas9 associated with modulator molecules (activators/repressors) guides regulatory molecules to the target locus and exerts specific functions in gene modulation, resulting in either gene expression inhibition or activation, without editing [57].

- Haploinsufficiency treatment:

The vast majority of genetic epilepsies are caused by loss-of-function mutations. dCas9 can increase the expression of the remaining functional wild-type allele and improve the phenotype by addressing haploinsufficiency. It is a non-specific approach that targets gene promoters generally and is effective for de novo mutations (a large proportion of epilepsy-causing mutations) [57].

The CRISPR system shows advantages and also challenges when used as an epigenetic modulator. Benefits that should be emphasized include precision targeting of specific genes, the reversible nature of modifications that avoid permanent DNA sequence changes, the ability to target multiple loci simultaneously, and the ability to modulate without overexpression effects. The drawbacks include delivery across blood–brain barrier, potential off-target effects, durability of epigenetic changes and immune responses to Cas9 protein. Moreover, studies for the safety and tolerability of CRISPR/CAS therapy are needed; to demonstrate effectiveness and evaluate possible adverse effects [88]. Despite an incomplete understanding of the mechanisms of the genetic epilepsy and current limitations of gene editing tools, CRISPR-mediated approaches have groundbreaking potential in the treatment of genetic epilepsy over the next decade. CRISPR can permanently alter the genetic code, offering potential for irreversible cures.

#### 3.9.4. Emerging Therapies—Antisense Oligonucleotides (ASO)

ASO are small (12–25 base pairs) synthetic sequences of DNA or RNA that can target specific RNAs with high selectivity.

Functional mechanisms: ASOs bind different RNA targets (especially mRNAs) via sequence complementarity. As a consequence, they block, diminish or modify RNA function, influencing the quantity of protein production or the quality of protein production (introducing new splicing sites). These molecules can be used to tackle diverse pathologies, including subtypes of genetic epilepsies.

**Applications in epilepsy**

- SCN2A Gain-of-Function:

A mouse model carrying the equivalent of the SCN2A mutated human gene was treated with ASOs, with positive results: reduced protein overexpression after intracerebral administration and reduced seizure activity [96].

- SCN1A Loss-of-Function (Dravet Syndrome):

ASO therapy was tested in a mouse model carrying the mutation, yielding positive results. ASO binds to the DNA sequence, removes the nonfunctional exon, and thereby increases the amount of functional mRNA, thereby increasing functional protein production [97].

Two trials on human patients, MONARCH and ADMIRAL, assessed the safety and efficacy of Zorevunersen (STK-001) in 81 patients aged 2–18 years. Zorevunersen is designed to upregulate the wild-type SCN1A allele in patients with Dravet syndrome. The treatment was safe and well-tolerated and showed a substantial reduction (>50%) in convulsive seizure frequency compared to classical antiseizure medication [98], representing significant clinical progress.

- SCN8A Mutations:

ASOs were tested in a mouse model, targeting the SCN8A loss-of-function mutation. The outcomes showed an increased survival rate [99].

- Temporal Lobe Epilepsy miRNAs:

Elevated levels of miR-134, miR-132, and miR-146a were targeted with antagonist ASO in preclinical studies, with positive outcomes in preclinical studies [100].

- lncRNAs Targeting:

lncRNAs upregulated or downregulated variants influence seizures in temporal lobe epilepsy, suggesting that lncRNAs can be targeted by ASOs with positive clinical results. BDNF-antisense RNA (lncRNA modulating brain-derived neurotrophic factor) can also be upregulated by ASO, with positive results in temporal lobe epilepsy [100].

- CircRNA Targeting:

circRNAs have binding activity to miRNAs. The blocking effect reduces pharmacoresistance to classical antiepileptic drugs. There are no current drugs addressing circRNAs, making the development of ASO therapy particularly important [100].

ASOs use has certain advantages and disadvantages. In terms of benefits, it can be mentioned that a high selectivity for specific mutations, both gain-of-function and loss-of-function mutations, can be addressed; also, splice site modulation (to remove non-functional exons) is feasible, along with miRNA inhibition (using antagomiRs) or natural antisense transcript blocking. One of the most important aspects of using ASOs in epilepsy is their characteristic of disease modification rather than symptom control. The challenges that need to be addressed include blood–brain barrier penetration, delivery methods (requires CNS administration for current ASOs), long-term durability of effects which are difficult to obtain in the epigenetic landscape, potential for off-target binding and finally, the cost of therapy.

Despite the novelty of the ASO therapies, the preclinical and clinical studies showed promising results of ASO as disease modification treatment. Epilepsy patients resistant to conventional treatment could become the first recipients of the ASO in the future, with increasing survival rates and better quality of life.

#### 3.9.5. Epigenetic Drug Development

Epigenetic drugs targets reversible gene expression regulators to treat neurological diseases by correcting abnormal gene activity without changing DNA. The most representative classes of epigenetic drugs are listed below:

A. DNA Methylation Modulators

First-generation DNMT inhibitors include 5-aza-cytidine and zebularine, causing global demethylation. They prevent DNMT from unbinding from DNA, and global alterations may contribute to neurological instability. More recently, second-generation DNMT inhibitors target the translational machinery of DNMT-encoding mRNAs, provide increased regulatory control, and hold the potential to ameliorate the development of epilepsy [46].

Adenosine Augmentation Therapy (AAT) is a methylation modulator that reverses DNA hypermethylation. It inhibits mossy fiber sprouting in the hippocampus and prevents progression of epilepsy for 3+ months through indirect methylation-mediated mechanisms [47].

The ketogenic diet is currently used as an epigenetic modulator. It consists of a low-carbohydrate, high-fat, and protein-restrictive diet and has shown anticonvulsant and potential antiepileptogenic effects, with positive effects in children with refractory epilepsy. Ketogenic diet induces metabolic changes, including augmentation of adenosine signaling, increased adenosine, and decreased DNA methylation in the pilocarpine model, reduces seizure burden, delays disease progression, and ameliorates DNA methylation-mediated alterations in gene expression [48].

B. HDAC Inhibitors

Valproic Acid is a well-established antiepileptic drug. Exhibit HDAC inhibitory activity plus ion channel effects: increases GABA levels and modulates epigenome through histone acetylation and DNA methylation. Chronic treatment results in increased H3 acetylation in the CNS and global DNA demethylation. The anticonvulsive effects are not fully attributed to epigenomic effects, but effectiveness increases over time (consistent with epigenetic mechanisms) [48].

Trichostatin A (TSA) prevents deacetylation at the GluA2 locus and increases basal acetylation and IEG expression, being an effective epigenetic agent for seizure suppression [47].

SAHA (Suberoylanilide Hydroxamic Acid) shows no anticonvulsant effects, but neuroprotective effects after status epilepticus. May improve cognitive deficits through reducing aberrant neurogenesis [47].

Sodium Butyrate increases H3/H4 acetylation in the hippocampus and cortex and has been shown to be effective in the electroconvulsive shock model of epileptogenesis.

Curcumin blocks acetylation, reduces histone modifications, and shows anticonvulsant effects.

Further research is needed for HDAC inhibitors. Current limitations for clinical use are the lack of selectivity of the current HDAC inhibitors, which target HDAC enzymes globally rather than specific post-translational modifications, and have off-target substrate effects. They require greater specificity for viable therapeutic use.

Future directions could focus on isoform-selective HDAC inhibitors, HDAC inhibitors that target specific histone readers or writers and combination therapies with traditional antiseizure medications.

### 3.10. Current Contradictions in Genetic Epilepsy Research

Understanding of genetic epilepsy has evolved from simple ion channel mutations to complex polygenic and epigenetic mechanisms, with future progress dependent on advanced sequencing, multi-omics analysis, and precision medicine approaches. Genetic epilepsy research shows contradictions across at least three main areas.

First, while earlier studies emphasized mutations in ion channel genes, recent studies report that genes involved in the mTOR pathway and synaptic function also contribute to the disease. DEPDC5, NPRL2, and NPRL3 (genes that encode proteins that downregulate mTOR signaling) demonstrated a clear role in the pathogenesis of epilepsy. Mutations in the GRIN2A gene, which encodes the protein subunits of the NMDA glutamate receptor (N-methyl-D-aspartate), can lead to a range of neurodevelopmental disorders, including epilepsy [101]. NMDA receptors are involved in excitatory synaptic transmission, plasticity, and excitotoxicity in the central nervous system.

Second, there are publications that contrast the traditional view of epilepsy as a primarily monogenic disorder with evidence that common forms are polygenic, involving both rare and common variants and leaving much heritability unexplained [1]. A GWAS study published in 2022 analyzed febrile seizures and concluded that common variants in genes that regulate the fever response and genes linked to seizure liability are involved in febrile seizures and account for 2.8% of susceptibility [102].

Third, there are studies that highlights importance of epigenetic modifications—such as DNA methylation—that may add to the complexity of epilepsy risk and treatment response. Chronic epilepsy status was associated with hypermethylation in central nervous system tissue, as shown by many studies [103]. These raise the possibility of DNMT (DNA methyltransferase) inhibitors as anti-epileptic drugs.

### 3.11. Future Directions and Emerging Approaches

High-throughput sequencing (exome and whole-genome methods) has already accelerated the discovery of rare and de novo variants. The trio-sequencing design (parents and child) can detect additional de novo variants in known genes or even identify new epilepsy-correlated genes. As NGS becomes more accessible, it is expected to lead to the discovery of new variants associated with epilepsy. Single-cell RNA sequencing is a novel analytical approach for brain tissue that can untangle the development of pathogenicity [104]. Large, well-phenotyped cohorts and advanced computational analysis are needed. Improved understanding of the genetic architecture of epilepsy will pave the way for the identification of new therapeutic targets.

Machine Learning Applications can provide predictive models for drug response based on methylation signatures. Patient stratification using epigenetic biomarkers is a future target, as is the integration of multidimensional data and the identification of convergent pathways across genetic backgrounds. As an example, DNA methylation features combined with clinical pathological factors improve the prediction of drug treatment response in TLE. Identification of genetic risk factors for drug response and side effects with clinical actionability (for example, human leukocyte antigen [HLA] genotyping) for antiepileptic drugs can contribute to decreasing adverse effects and improving outcomes.

Detection of somatic variants causative of epilepsy is an emerging approach in the genetic characterization of epilepsy. The somatic mutations in the mTOR pathway encoding genes (TSC1, TSC2, MTOR, PIK3CA, AKT3, DEPDC5) limited to the brain tissue were identified in focal epilepsies. Future studies will presumably identify more patients carrying somatic pathogenic mutations, both as isolated variants or associated somatic and germline variants [101].

Data sharing and international collaboration are crucial for expanding genetic discovery. Harmonization of data standards and ethical/legal frameworks is an important step toward facilitating cooperation, which can lead to increased statistical power, the discovery of rare variants, and the global applicability of such a coordinated effort.

**Multi-Omics Integration**

Multi-omics integration is a holistic approach that combines data from multiple ‘omic’ layers to create a comprehensive understanding of complex biological systems, revealing interactions and interconnected pathways for better diagnosis, prognosis, and treatment. Comprehensive profiling results from integration of multiple data layers:

- Genomics: whole-genome sequencing, exome sequencing;
- Epigenomics: methylation profiling (methyl-seq, arrays), ChIP-seq, ATAC-seq;
- Transcriptomics: RNA-seq for coding and non-coding RNAs;
- Proteomics: protein expression and modification;
- Metabolomics: metabolic pathway analysis.

**Personalized Treatment Paradigm**

A personalized treatment paradigm (precision medicine) transforms healthcare from a one-size-fits-all approach to tailoring prevention, diagnosis, and treatment to each patient. This patient-centered model applies to the diagnostic phase, therapeutic selection, and monitoring and adjustment:

The diagnostic phase includes genetic sequencing to identify causative variants, epigenetic profiling (methylation, histone marks, ncRNA), functional validation in patient-derived iPSCs, and specific biomarker identification.

Therapeutic selection should rely on genotype-matched treatments (e.g., sodium channel blockers for specific SCN variants), epigenetic modulators based on methylation and acetylation status, combination therapies targeting genetic and epigenetic layers, and antagomiRs for specific miRNA dysregulation.

The monitoring and adjustment phase considers circulating epigenetic biomarkers for treatment response, adjusting epigenetic interventions based on evolving profiles, and implementing prevention strategies during the latent period.

## 4. Discussions

Understanding of genetic epilepsy has evolved from simple ion channel mutations to complex polygenic and epigenetic mechanisms, with future progress dependent on advanced sequencing, multi-omics analysis, and precision medicine approaches.

Epilepsy syndromes rooted in genetics present a varied and complex array of disorders, posing profound challenges for those affected and their families. Progress in genetic research has significantly deepened our comprehension of these syndromes, facilitating more precise diagnoses and personalized treatment approaches. Continuous research and interdisciplinary cooperation involving genetic specialists, neurologists, and healthcare experts are crucial for advancing outcomes for individuals grappling with genetic epilepsy syndromes. As our understanding broadens, the aim is to forge ahead towards more potent therapies and, ultimately, potential cures for these intricate conditions.

The molecular landscape of genetic epilepsy syndromes is multifaceted, with pathways that intersect to regulate excitability, development, and synaptic function [105]. While targeted therapies—such as mTOR inhibitors and gene-specific treatments—are emerging, a deeper understanding of pathway crosstalk and compensatory mechanisms is needed. Genetic epilepsy involves complex interactions between excitatory/inhibitory homeostasis (ion channels, GABAergic), epigenetic plasticity and induction (mTOR, chromatin remodeling), fundamental cellular processes (autophagy, senescence, inflammation, MAPK/JNK), and novel genetic targets (CACNA1E, WDR26, SYNGAP1, zinc finger factors, and chromatin remodeling). An integrated systemic approach, combining mTOR/autophagy modulation, epigenetic control, reduction in molecular senescence, and ion channel corrections, is promising for the future in the therapy of complex epilepsies.

Traditional pathways (channelopathies, GABA) remain a foundation but intersect with superordinate signaling (mTOR, epigenetics, autophagy). Chromatin remodeling factors and zinc fingers are potential new players, opening new avenues of research. Senescence signaling (MAPK/JNK) and inflammation have become emerging therapeutic targets.

Future research should focus on mapping interactions among pathways to identify convergence points for therapy, clarifying genotype–phenotype relationships, especially in cases of variable expressivity, exploring epigenetic modulation as a therapeutic strategy, and, finally, developing personalized interventions based on molecular profiling.

This integrative approach may pave the way for precision medicine in epilepsy, transforming our ability to predict, prevent, and treat these complex disorders.

## 5. Conclusions

Epilepsy poses significant challenges in healthcare due to its substantial financial burdens and societal impacts. These factors underscore the urgent need for deeper investigation into the disorder’s genetic and molecular foundations.

Modern medicine is increasingly embracing tailored and individualized strategies. This shift demands extensive research and comprehensive data collection to construct distinct patient profiles. Such initiatives promise greater precision in diagnosis and treatment.

Lowering the costs of NGS techniques (WGS or WES) enabled faster, more precise identification of rare genetic variants and consistent progress in understanding epileptic conditions and neurologic diseases [104].

The therapeutic management of epileptic seizures remains a challenge. Many currently available medications are ineffective and cause undesirable side effects. To improve treatment success, researchers aim to gather more information on epilepsy to develop individualized treatment plans with minimal side effects. This approach requires a more precise diagnosis, which can be achieved through improved characterization of the various epilepsies [106].

Recent studies showed that the genetic landscape of epilepsy is broader and more complex than previously thought, suggesting a shift from single-gene explanations toward integrated models of diverse genetic and epigenetic contributors.

In conclusion, the convergence of genetic mutations and epigenetic dysregulation defines the complex landscape of epilepsy syndromes. While ion channelopathies and signaling pathway disruptions remain central to epileptogenesis, epigenetic mechanisms—such as chromatin remodeling, histone modifications, and DNA methylation—emerge as equally critical determinants of disease expression and variability. These insights not only expand our understanding of epilepsy pathophysiology but also open translational opportunities: epigenetic biomarkers for diagnosis, epigenome-targeted therapies for precision medicine, and CRISPR/dCas9-based editing for long-term disease modification. Future research should prioritize integrated multi-omics approaches that capture both genetic architecture and epigenetic states, enabling a systems-level view of epileptogenesis. By reframing epilepsy as an epigenetically regulated disorder, we can accelerate the development of innovative therapeutic strategies and improve outcomes for patients with refractory syndromes. Continued investment in research, technology, and local studies will pave the way for more effective, individualized care. Ultimately, this approach holds the promise of transforming epilepsy management and enhancing patients’ quality of life.

Although current literature offers a substantial amount of information about the genetic foundations of epilepsy, there are still considerable gaps in our understanding. These gaps relate to the intricate interaction between genetic and environmental factors, the discovery of new genes associated with epilepsy susceptibility, and the clarification of the specific functional impacts of these genetic variants [106]. Further research to address these knowledge gaps is crucial for advancing our understanding of the diverse etiological pathways that contribute to epilepsy.

## Figures and Tables

**Table 1:** Clinical and Therapeutic Implications of Ion Channels.

Therapeutic Strategy	Channel Target	Mode of Action	Clinical Status
Sodium channel blockers	SCN2A GOF	Reduce persistent Na+ current	Phase II trials [15]
Kv1.1 gene therapy	KCNA1	Enhance repolarization	Preclinical [10]
GABA_A potentiators	GABRA1 LOF	Enhance inhibition	Clinical use [16]
Chloride homeostasis modulators	CLCN2, KCC2	Restore GABAergic inhibition	Experimental [17]
CRISPR/CAS9 correction	SCN1A	Gene editing	Preclinical (iPSC, mouse models) [18]

Kv1.1 = voltage-gated potassium channel that is part of the Kv channel family. GABA_A = γ-aminobutyric acid type A receptors. iPSC = induced pluripotent stem cells.

**Table 2:** Clinical and Therapeutic Implications of mTOR signaling pathways.

Strategy	Molecular Target	Example
mTOR inhibition	mTORC1	Everolimus (for TSC), Sirolimus [25]
Autophagy enhancement	ULK1, AMPK	Metformin (preclinical), Trehalose [26]
Somatic mutation targeting	mTOR, DEPDC5	Region-specific gene editing (in vivo CRISPR, preclinical) [27]
Precision diagnostics	Deep brain sequencing	FCD lesion genotyping, mosaicism detection [28]

FCD = focal cortical dysplasia.

**Table 3:** Therapeutic Implications of the GABAergic Signaling Pathway.

Target	Strategy	Example
GABA_A receptor	Positive allosteric modulators	Benzodiazepines, neurosteroids [35]
Chloride transport	NKCC1 inhibition/KCC2 enhancement	Bumetanide (limited CNS efficacy) [36]
Gene therapy	GABRA1/GABRG2 rescue	In preclinical development [37]
Interneuron protection	SCN1A gene therapy or interneuron transplantation	Experimental models of Dravet syndrome [38]

**Table 4:** Therapeutic implications of MAPK/JNK-mediated neuronal senescence in epilepsy.

Therapeutic Target	Approach	Status
JNK Inhibition	Small molecule inhibitors (e.g., SP600125)	Preclinical models [43]
Senolytics	Agents selectively eliminating senescent neurons	Emerging research [44]
Anti-inflammatory	Targeting SASP components	Clinical trials in neurodegeneration [45]

**Table 5:** Altered histone modification patterns.

Model	Modification	Effect	Reference Gene
SE Pilo Rat	↓ H4 Acetylation	↓ GluR2/Gria2 expression	GRIA2
SE KA Mouse	↑ H3 Phosphorylation, ↑ H4 Acetylation	c-Fos induction	FOS
SE KA Mouse	↑ HDAC5	↓ H3 and H4 Acetylation	Multiple
TLE Patient/SE Pilo Rat	↑ HDAC2	Enhanced neuronal excitability	Synaptic plasticity genes

SE = status epilepticus; KA = kainic acid; TLE = temporal lobe epilepsy; ↑ = increase; ↓ = decrease

**Table 6:** Therapeutic perspectives of epigenetic mechanisms in epileptogenesis.

Strategy	Target	Status
HDAC inhibitors	Histone acetylation	Preclinical/clinical trials [52]
Epigenetic editing	CRISPR-dCas9-based chromatin modulation	Experimental [57]
Small molecules modulating ZNF activity	Transcription factor modulation	Early-stage research [58]

**Table 7:** Inheritance Patterns in Epilepsies.

Inheritance Patterns in Epilepsies	Examples of Corresponding Genetic Diseases
**Mendelian Inheritance**	
AD	BFNE, ADNFLE
AR	Lafora disease, Unverricht–Lundborg disease
XLR	ARX-associated epilepsies
XLD	double cortex syndrome, periventricular nodular heterotopia
XL with male sparing	PCDH19-related epilepsy
**Non-mendelian Inheritance**	Most cases of epilepsy

ADNFLE = autosomal dominant nocturnal frontal lobe epilepsy; BFNE = benign familial neonatal epilepsy.

**Table 8:** Genes Involved in Monogenic Epilepsies.

Examples of Genes Involved in Monogenic Epilepsies	Mechanism and Function	Relevant Corresponding Phenotypes
ARX	Encodes the aristaless-related homeobox protein, involved in cerebral development [67]	DEE1
CHRNA4	Encodes the α4 subunit of the α4β2 nAChR receptor, involved in neurotransmitter release and synaptic signaling [68]	ADNFLE
CHRNB2	Encodes the β2 subunit of the α4β2 nAChR receptor, involved in neurotransmitter release and synaptic signaling [68]	ADNFLE
DEPDC5	Encodes a DEP domain-containing protein, part of the GATOR1 complex, which inhibits the mTOR pathway [69]	Familial focal epilepsy with variable foci, DEE111
GABRG2	Encodes the γ2 subunit of α1β2γ2 GABA_A_R, involved in the inhibitory neurotransmission in the brain [70]	DEE74, GEFS+
KCNQ2	Encodes a subunit of the voltage-gated potassium channel in the brain, underlying the M-current, which plays a crucial role in regulating neuronal excitability [71]	BFNE, DEE7
KCNQ3	Encodes a subunit of the voltage-gated potassium channel in the brain, underlying the M-current, which plays a crucial role in regulating neuronal excitability [71]	BFNE
KCNT1	Encodes a sodium-activated potassium channel widely expressed in the nervous system, involved in processes such as neuronal excitability, delayed outward currents, and brain development [72]	Malignant migrating partial seizures of infancy (DEE14), ADNFLE
LGI1	Encodes a secreted leucine-rich protein expressed in the central nervous system, involved in the development of glutamatergic synapses and regulation of voltage-gated potassium channels [73]	Familial lateral temporal lobe epilepsy
PRRT2	Encodes a presynaptic membrane protein expressed in the central nervous system, important in neurotransmitter release [74]	Benign familial infantile seizures
SCN1A	Encodes the α subunit of the NaV1.1 voltage-gated sodium channel [75]. Voltage-gated sodium channels are essential for the initiation and propagation of action potentials in excitable membranes. The structure of sodium channels includes a pore-forming α subunit, responsible for channel activity, and two β subunits, which modulate channel kinetics [76]	Dravet syndrome (DEE6A), GEFS+
SCN2A	Encodes the α subunit of the NaV1.2 voltage-gated sodium channel [77]	Benign familial infantile seizures, DEE11
SCN1B	Encodes the β-1 subunit of voltage-gated sodium channels [77]	GEFS+, DEE52
STXBP1	Encodes a syntaxin-binding protein expressed in neurons, involved in the release of neurotransmitters in the brain [78]	DEE4

ADNFLE = autosomal dominant nocturnal frontal lobe epilepsy; BFNE = benign familial neonatal epilepsy, DEE = developmental and epileptic encephalopathy; GABA_A_R = gamma-aminobutyric acid type A receptor; GATOR1 = GTPase-activating protein activity toward Rag 1; GEFS+ = genetic epilepsy with febrile seizures plus; mTOR = mechanistic target of rapamycin.

## Data Availability

No new data were created or analyzed in this study. Data sharing is not applicable to this article.

## References

[1] Ruggiero S.M., Xian J., Helbig I. (2023). The current landscape of epilepsy genetics: Where are we, and where are we going?. Curr. Opin. Neurol..

[2] Manokaran R.K., Sharma S., Ramachandrannair R. (2024). The 2022 International League against Epilepsy Classification and Definition of Childhood Epilepsy Syndromes: An Update for Pediatricians. Indian Pediatr..

[3] Thakran S., Guin D., Singh P., Singh P., Kukal S., Rawat C., Yadav S., Kushwaha S.S., Srivastava A.K., Hasija Y. (2020). Genetic Landscape of Common Epilepsies: Advancing towards Precision in Treatment. Int. J. Mol. Sci..

[4] Joshi S., Lal D., Haneef Z. (2024). Genetics of Epilepsy. Epilepsy Fundamentals.

[5] Wang J., Ou S.W., Wang Y.J. (2017). Distribution and function of voltage-gated sodium channels in the nervous system. Channels.

[6] Steinlein O.K. (2008). Genetics and Epilepsy. Epilepsy Psychiatry.

[7] Brunklaus A., Brünger T., Feng T., Fons C., Lehikoinen A., Panagiotakaki E., Vintan M.A., Symonds J., Andrew J., Arzimanoglou A. (2022). The gain of function SCN1A disorder spectrum: Novel epilepsy phenotypes and therapeutic implications. Brain.

[8] Zhao T., Wang L., Chen F. (2024). Potassium channel-related epilepsy: Pathogenesis and clinical features. Epilepsia Open.

[9] Narayanan D.L., Somashekar P.H., Majethia P., Shukla A. (2022). KCTD7-related progressive myoclonic epilepsy: Report of three Indian families and review of literature. Clin. Dysmorphol..

[10] Snowball A., Chabrol E., Wykes R.C., Shekh-Ahmad T., Cornford J.H., Lieb A., Hughes M.P., Massaro G., Rahim A.A., Hashemi K.S. (2019). Epilepsy Gene Therapy Using an Engineered Potassium Channel. J. Neurosci..

[11] Szymanowicz O., Drużdż A., Słowikowski B., Pawlak S., Potocka E., Goutor U., Konieczny M., Ciastoń M., Lewandowska A., Jagodziński P.P. (2024). A Review of the CACNA Gene Family: Its Role in Neurological Disorders. Diseases.

[12] Cho Y.S., Kim Y.S., Moozhayil S.J., Yang E.S., Bae Y.C. (2015). The expression of hyperpolarization-activated cyclic nucleotide-gated channel 1 (HCN1) and HCN2 in the rat trigeminal ganglion, sensory root, and dental pulp. Neuroscience.

[13] Galanopoulou A.S. (2010). Mutations affecting GABAergic signaling in seizures and epilepsy. Pflügers Arch. Eur. J. Physiology.

[14] Stöber T.M., Batulin D., Triesch J., Narayanan R., Jedlicka P. (2023). Degeneracy in epilepsy: Multiple routes to hyperexcitable brain circuits and their repair. Commun. Biol..

[15] Meisler M.H., Hill S.F., Yu W. (2021). Sodium channelopathies in neurodevelopmental disorders. Nat. Rev. Neurosci..

[16] Perucca E., White H.S., Bialer M. (2023). New GABA-Targeting Therapies for the Treatment of Seizures and Epilepsy: II. Treatments in Clinical Development. CNS Drugs.

[17] Ni M.M., Sun J.Y., Li Z.Q., Qiu J.C., Wu C.F. (2025). Role of voltage-gated chloride channels in epilepsy: Current insights and future directions. Front. Pharmacol..

[18] Yamagata T., Raveau M., Kobayashi K., Miyamoto H., Tatsukawa T., Ogiwara I., Itohara S., Hensch T.K., Yamakawa K. (2020). CRISPR/dCas9-based Scn1a gene activation in inhibitory neurons ameliorates epileptic and behavioral phenotypes of Dravet syndrome model mice. Neurobiol. Dis..

[19] Limanaqi F., Biagioni F., Busceti C.L., Fabrizi C., Frati A., Fornai F. (2020). mTOR-Related Cell-Clearing Systems in Epileptic Seizures, an Update. Int. J. Mol. Sci..

[20] Guerrini R., Cavallin M., Pippucci T., Rosati A., Bisulli F., Dimartino P., Barba C., Garbelli R., Buccoliero A.M., Tassi L. (2020). Is Focal Cortical Dysplasia/Epilepsy Caused by Somatic *MTOR* Mutations Always a Unilateral Disorder?. Neurol. Genet..

[21] Ali N.H., Al-Kuraishy H.M., Al-Gareeb A.I., Alnaaim S.A., Alexiou A., Papadakis M., Saad H.M., Batiha G.E. (2023). Autophagy and autophagy signaling in Epilepsy: Possible role of autophagy activator. Mol. Med..

[22] Baldassari S., Klingler E., Teijeiro L.G., Doladilhe M., Raoux C., Roig-Puiggros S., Bizzotto S., Couturier J., Gilbert A., Sami L. (2025). Single-cell genotyping and transcriptomic profiling of mosaic focal cortical dysplasia. Nat. Neurosci..

[23] Pfisterer U., Petukhov V., Demharter S., Meichsner J., Thompson J.J., Batiuk M.Y., Asenjo-Martinez A., Vasistha N.A., Thakur A., Mikkelsen J. (2020). Identification of epilepsy-associated neuronal subtypes and gene expression underlying epileptogenesis. Nat. Commun..

[24] Moloney P.B., Cavalleri G.L., Delanty N. (2021). Epilepsy in the mTORopathies: Opportunities for precision medicine. Brain Commun..

[25] Śmiałek D., Kotulska K., Duda A., Jóźwiak S. (2023). Effect of mTOR Inhibitors in Epilepsy Treatment in Children with Tuberous Sclerosis Complex Under 2 Years of Age. Neurol. Ther..

[26] Thellung S., Corsaro A., Nizzari M., Barbieri F., Florio T. (2019). Autophagy Activator Drugs: A New Opportunity in Neuroprotection from Misfolded Protein Toxicity. Int. J. Mol. Sci..

[27] Boff M.O., Xavier F.A.C., Diz F.M., Gonçalves J.B., Ferreira L.M., Zambeli J., Pazzin D.B., Previato T.T.R., Erwig H.S., Gonçalves J.I.B. (2025). mTORopathies in Epilepsy and Neurodevelopmental Disorders: The Future of Therapeutics and the Role of Gene Editing. Cells.

[28] Garcia C.A.B., Zubair M., Santos M.V., Lee S.H., Graham I.A., Stanley V., George R.D., Gleeson J.G., Machado H.R., Yang X. (2025). Identification of Novel Mosaic Variants in Focal Epilepsy-Associated Patients’ Brain Lesions. Genes.

[29] Leitch B. (2024). Parvalbumin Interneuron Dysfunction in Neurological Disorders: Focus on Epilepsy and Alzheimer’s Disease. Int. J. Mol. Sci..

[30] Lange M., Jüngling K., Paulukat L., Vieler M., Gaburro S., Sosulina L., Blaesse P., Sreepathi H.K., Ferraguti F., Pape H.C. (2014). Glutamic Acid Decarboxylase 65: A Link Between GABAergic Synaptic Plasticity in the Lateral Amygdala and Conditioned Fear Generalization. Neuropsychopharmacology.

[31] Platzer K., Sticht H., Bupp C., Ganapathi M., Pereira E.M., Le Guyader G., Bilan F., Henderson L.B., Lemke J.R., Taschenberger H. (2022). De Novo Missense Variants in SLC32A1 Cause a Developmental and Epileptic Encephalopathy Due to Impaired GABAergic Neurotransmission. Ann. Neurol..

[32] Parenti I., Leitão E., Kuechler A., Villard L., Goizet C., Courdier C., Bayat A., Rossi A., Julia S., Bruel A.L. (2022). The different clinical facets of *SYN1*-related neurodevelopmental disorders. Front. Cell Dev. Biol..

[33] Kang J.Q. (2017). Defects at the crossroads of GABAergic signaling in generalized genetic epilepsies. Epilepsy Res..

[34] Sutula T. (2002). Seizure-Induced Axonal Sprouting: Assessing Connections Between Injury, Local Circuits, and Epileptogenesis. Epilepsy Curr..

[35] Vanover K.E., Suruki M., Robledo S., Huber M., Wieland S., Lan N.C., Gee K.W., Wood P.L., Carter R.B. (1999). Positive allosteric modulators of the GABA(A) receptor: Differential interaction of benzodiazepines and neuroactive steroids with ethanol. Psychopharmacol..

[36] Löscher W., Kaila K. (2022). CNS pharmacology of NKCC1 inhibitors. Neuropharmacology.

[37] Turner T.J., Zourray C., Schorge S., Lignani G. (2021). Recent advances in gene therapy for neurodevelopmental disorders with epilepsy. J. Neurochem..

[38] Mich J.K., Ryu J., Wei A.D., Gore B.B., Guo R., Bard A.M., Martinez R.A., Luber E.M., Liu J., Bishaw Y.M. (2025). Interneuron-specific dual-AAV SCN1A gene replacement corrects epileptic phenotypes in mouse models of Dravet syndrome. Sci. Transl. Med..

[39] Galosi S., Pollini L., Novelli M., Bernardi K., Di Rocco M., Martinelli S., Leuzzi V. (2022). Motor, epileptic, and developmental phenotypes in genetic disorders affecting G protein coupled receptors-cAMP signaling. Front. Neurol..

[40] Liu S., Luo Z., Li F., Zhang L., Xie M., Yang J., Xu Z. (2024). The JNK Signaling Pathway Regulates Seizures Through ENT1 in Pilocarpine-Induced Epilepsy Rat Model. CNS Neurosci. Ther..

[41] Gong X., Lu W., Xiao Q., Wang X., Cui C., Tang H. (2025). Construction of epilepsy diagnosis model based on cell senescence-related genes and its potential mechanism. Front. Neurol..

[42] Zhang W., Wang X., Yu M., Li J.A., Meng H. (2018). The c-Jun N-terminal kinase signaling pathway in epilepsy: Activation, regulation, and therapeutics. J. Recept. Signal Transduct. Res..

[43] Busquets O., Ettcheto M., Cano A.R., Manzine P., Sánchez-Lopez E., Espinosa-Jiménez T., Verdaguer E., Dario Castro-Torres R., Beas-Zarate C.X., Sureda F. (2019). Role of c-Jun N-Terminal Kinases (JNKs) in Epilepsy and Metabolic Cognitive Impairment. Int. J. Mol. Sci..

[44] Khan T., Hussain A.I., Casilli T.P., Frayser L., Cho M., Williams G., McFall D., Forcelli P.A. (2024). Prophylactic senolytic treatment in aged mice reduces seizure severity and improves survival from Status Epilepticus. Aging Cell.

[45] Wang Y., Kuca K., You L., Nepovimova E., Heger Z., Valko M., Adam V., Wu Q., Jomova K. (2024). The role of cellular senescence in neurodegenerative diseases. Arch. Toxicol..

[46] Conboy K., Henshall D.C., Brennan G.P. (2021). Epigenetic principles underlying epileptogenesis and epilepsy syndromes. Neurobiol. Dis..

[47] Henshall D.C., Kobow K. (2015). Epigenetics and Epilepsy. Cold Spring Harb. Perspect. Med..

[48] Li Y., Su Z., Zhao K., Liu X., Chen S., Yang X., Zhou L. (2025). Epigenetic Mechanisms in the Pathophysiology and Progression of Epilepsy: A Comprehensive Review of Experimental and Clinical Studies. Curr. Neuropharmacol..

[49] Khurana I., Khoury J., Busch R., Blümcke I., Najm I., El-Osta A. (2025). Paired blood and brain tissue methylation biomarkers in focal cortical dysplasia. Brain Commun..

[50] Gates L.A., Shi J., Rohira A.D., Feng Q., Zhu B., Bedford M.T., Sagum C.A., Jung S.Y., Qin J., Tsai M.J. (2017). Acetylation on histone H3 lysine 9 mediates a switch from transcription initiation to elongation. J. Biol. Chem..

[51] Nasrallah K., Frechou M.A., Yoon Y.J., Persaud S., Gonçalves J.T., Castillo P.E. (2022). Seizure-induced strengthening of a recurrent excitatory circuit in the dentate gyrus is proconvulsant. Proc. Natl. Acad. Sci. USA.

[52] Zhou C., Zhao D., Wu C., Wu Z., Zhang W., Chen S., Zhao X., Wu S. (2024). Role of histone deacetylase inhibitors in non-neoplastic diseases. Heliyon.

[53] Tang L., Nogales E., Ciferri C. (2010). Structure and function of SWI/SNF chromatin remodeling complexes and mechanistic implications for transcription. Prog. Biophys. Mol. Biol..

[54] Galizia E.C., Myers C.T., Leu C., de Kovel C.G., Afrikanova T., Cordero-Maldonado M.L., Martins T.G., Jacmin M., Drury S., Krishna Chinthapalli V. (2015). CHD2 variants are a risk factor for photosensitivity in epilepsy. Brain.

[55] Proietti J., Amadori E., Striano P., Ricci E., Cordelli D.M., Bana C., Dilena R., Gardella E., Klint Nielsen J.E., Pisani F. (2021). Epilepsy features in ARID1B-related Coffin-Siris syndrome. Epileptic Disord..

[56] Poeta L., Padula A., Lioi M.B., van Bokhoven H., Miano M.G. (2021). Analysis of a Set of KDM5C Regulatory Genes Mutated in Neurodevelopmental Disorders Identifies Temporal Coexpression Brain Signatures. Genes.

[57] Policarpi C., Dabin J., Hackett J.A. (2021). Epigenetic editing: Dissecting chromatin function in context. Bioessays.

[58] Koehler A.N. (2010). A complex task? Direct modulation of transcription factors with small molecules. Curr. Opin. Chem. Biol..

[59] Guerri G., Castori M., D’Agruma L., Petracca A., Kurti D., Bertelli M. (2020). Genetic Analysis of Genes Associated with Epilepsy. Acta Bio-Medica Atenei Parm..

[60] Helbig I. (2015). Genetic Causes of Generalized Epilepsies. Semin. Neurol..

[61] Berkovic S.F. (2015). Genetics of Epilepsy in Clinical Practice. Epilepsy Curr..

[62] Herghelegiu C.G., Veduta A., Stefan M.F., Magda S.L., Ionascu I., Rădoi V.E., Oprescu D.N., Calin A.M. (2023). Hyperglycosylated-hCG: Its Role in Trophoblast Invasion and Intrauterine Growth Restriction. Cells.

[63] Giucă A., Mitu C., Popescu B.O., Bastian A.E., Capşa R., Mursă A., Rădoi V., Popescu B.A., Jurcuţ R. (2020). Novel FHL1 mutation variant identified in a patient with nonobstructive hypertrophic cardiomyopathy and myopathy—A case report. BMC Med. Genet..

[64] Depienne C., LeGuern E. (2012). PCDH19-Related Infantile Epileptic Encephalopathy: An Unusual X-Linked Inheritance Disorder. Hum. Mutat..

[65] Arsov T., Mullen S.A., Rogers S., Phillips A.M., Lawrence K.M., Damiano J.A., Goldberg-Stern H., Afawi Z., Kivity S., Trager C. (2012). Glucose Transporter 1 Deficiency in the Idiopathic Generalized Epilepsies. Ann. Neurol..

[66] Dibbens L.M., de Vries B., Donatello S., Heron S.E., Hodgson B.L., Chintawar S., Crompton D.E., Hughes J.N., Bellows S.T., Klein K.M. (2013). Mutations in DEPDC5 Cause Familial Focal Epilepsy with Variable Foci. Nat. Genet..

[67] Mastrangelo M., Leuzzi V. (2012). Genes of early-onset epileptic encephalopathies: From genotype to phenotype. Pediatr. Neurol..

[68] Jalaiei A., Asadi M.R., Daneshmandpour Y., Rezazadeh M., Ghafouri-Fard S. (2025). Clinical, molecular, physiologic, and therapeutic feature of patients with CHRNA4 and CHRNB2 deficiency: A systematic review. J. Neurochem..

[69] Baldassari S., Licchetta L., Tinuper P., Bisulli F., Pippucci T. (2016). GATOR1 complex: The common genetic actor in focal epilepsies. J. Med. Genet..

[70] Shen D., Hernandez C.C., Shen W., Hu N., Poduri A., Shiedley B., Rotenberg A., Datta A.N., Leiz S., Patzer S. (2017). De novo GABRG2 mutations associated with epileptic encephalopathies. Brain.

[71] Wang H.S., Pan Z., Shi W., Brown B.S., Wymore R.S., Cohen I.S., Dixon J.E., McKinnon D. (1998). KCNQ2 and KCNQ3 potassium channel subunits: Molecular correlates of the M-channel. Science.

[72] Zhang J., Liu S., Fan J., Yan R., Huang B., Zhou F., Yuan T., Gong J., Huang Z., Jiang D. (2023). Structural basis of human Slo2.2 channel gating and modulation. Cell Rep..

[73] Anderson M.P. (2010). Arrested glutamatergic synapse development in human partial epilepsy. Epilepsy Curr..

[74] Li Y., Chen S., Wang C., Wang P., Li X., Zhou L. (2021). PRRT2 gene and protein in human: Characteristics, evolution and function. Acta Epileptol..

[75] Lossin C., Rhodes T.H., Desai R.R., Vanoye C.G., Wang D., Carniciu S., Devinsky O., George A.L. (2003). Epilepsy-associated dysfunction in the voltage-gated neuronal sodium channel SCN1A. J. Neurosci..

[76] Bouza A.A., Isom L.L. (2018). Voltage-Gated Sodium Channel β Subunits and Their Related Diseases. Handb. Exp. Pharmacol..

[77] Ademuwagun I.A., Rotimi S.O., Syrbe S., Ajamma Y.U., Adebiyi E. (2021). Voltage Gated Sodium Channel Genes in Epilepsy: Mutations, Functional Studies, and Treatment Dimensions. Front. Neurol..

[78] STXBP1 Encephalopathy with Epilepsy. https://www.ncbi.nlm.nih.gov/books/NBK396561/.

[79] Oliver K.L., Ellis C.A., Scheffer I.E., Ganesan S., Leu C., Sadleir L.G., Heinzen E.L., Mefford H.C., Bass A.J., Curtis S.W. (2022). Common Risk Variants for Epilepsy Are Enriched in Families Previously Targeted for Rare Monogenic Variant Discovery. eBioMedicine.

[80] Stenhouse S.A., Ellis R., Zuberi S. (2013). SCN1A Genetic Test for Dravet Syndrome (Severe Myoclonic Epilepsy of Infancy and its Clinical Subtypes) for use in the Diagnosis, Prognosis, Treatment and Management of Dravet Syndrome. PLoS Curr..

[81] Oyrer J., Maljevic S., Scheffer I.E., Berkovic S.F., Petrou S., Reid C.A. (2018). Ion Channels in Genetic Epilepsy: From Genes and Mechanisms to Disease-Targeted Therapies. Pharmacol. Rev..

[82] Dowa Y., Shiihara T., Akiyama T., Hasegawa K., Inoue F., Watanabe M. (2020). A Case of Pyridoxine-Dependent Epilepsy with Novel ALDH7A1 Mutations. Oxf. Med. Case Rep..

[83] Anwar A., Saleem S., Patel U.K., Arumaithurai K., Malik P. (2019). Dravet Syndrome: An Overview. Cureus.

[84] Asadi-Pooya A.A. (2017). Lennox-Gastaut Syndrome: A Comprehensive Review. Neurol. Sci..

[85] Kessler S.K., McGinnis E. (2019). A Practical Guide to Treatment of Childhood Absence Epilepsy. Pediatr. Drugs.

[86] Pavone P., Polizzi A., Marino S.D., Corsello G., Falsaperla R., Marino S., Ruggieri M. (2020). West Syndrome: A Comprehensive Review. Neurol. Sci..

[87] Myers K.A. (2022). Genetic Epilepsy Syndromes. Contin. Lifelong Learn. Neurol..

[88] Carpenter J.C., Lignani G. (2021). Gene Editing and Modulation: The Holy Grail for the Genetic Epilepsies?. Neurotherapeutics.

[89] Colasante G., Qiu Y., Massimino L., Di Berardino C., Cornford J.H., Snowball A., Weston M., Jones S.P., Giannelli S., Lieb A. (2020). In vivo CRISPRa decreases seizures and rescues cognitive deficits in a rodent model of epilepsy. Brain.

[90] Liu J., Gao C., Chen W., Ma W., Li X., Shi Y., Zhang H., Zhang L., Long Y., Xu H. (2016). CRISPR/CAS9 facilitates investigation of neural circuit disease using human iPSCs: Mechanism of epilepsy caused by an SCN1A loss-of-function mutation. Transl. Psychiatry.

[91] Voskobiynyk Y., Battu G., Felker S.A., Cochran J.N., Newton M.P., Lambert L.J., Kesterson R.A., Myers R.M., Cooper G.M., Roberson E.D. (2021). Aberrant regulation of a poison exon caused by a non-coding variant in a mouse model of Scn1a-associated epileptic encephalopathy. PLoS Genet..

[92] Møller R.S., Weber Y.G., Klitten L.L., Trucks H., Muhle H., Kunz W.S., Mefford H.C., Franke A., Kautza M., Wolf P. (2013). EPICURE Consortium. Exon-disrupting deletions of NRXN1 in idiopathic generalized epilepsy. Epilepsia.

[93] Wagnon J.L. (2020). Promoting CRISPRa for Targeted Treatment of Epilepsy. Epilepsy Curr..

[94] Doudna J.A. (2020). The promise and challenge of therapeutic genome editing. Nature.

[95] Liao H.K., Hatanaka F., Araoka T., Reddy P., Wu M.Z., Sui Y., Yamauchi T., Sakurai M., O’Keefe D.D., Núñez-Delicado E. (2017). In Vivo Target Gene Activation via CRISPR/CAS9-Mediated Trans-epigenetic Modulation. Cell.

[96] Li M., Jancovski N., Jafar-Nejad P., Burbano L.E., Rollo B., Richards K., Drew L., Sedo A., Heighway J., Pachernegg S. (2021). Antisense oligonucleotide therapy reduces seizures and extends life span in an SCN2A gain-of-function epilepsy model. J. Clin. Investig..

[97] Han Z., Chen C., Christiansen A., Ji S., Lin Q., Anumonwo C., Liu C., Leiser S.C., Meena Aznarez I., Liau G. (2020). Antisense oligonucleotides increase Scn1a expression and reduce seizures and SUDEP incidence in a mouse model of Dravet syndrome. Sci. Transl. Med..

[98] (2024). Stoke Therapeutics Presents Zorevunersen Data Showing Substantial Reductions in Seizures and Improvements in Multiple Measures of Cognition and Behavior That Support the Potential for Disease Modification in Dravet Syndrome. News release. Stoke Therapeutics.

[99] Lenk G.M., Jafar-Nejad P., Hill S.F., Huffman L.D., Smolen C.E., Wagnon J.L., Petit H., Yu W., Ziobro J., Bhatia K. (2020). Scn8a Antisense Oligonucleotide Is Protective in Mouse Models of SCN8A Encephalopathy and Dravet Syndrome. Ann. Neurol..

[100] Stephen J.W.G., Stephen J.G., Ali M., Kumar A., Jose S. (2022). Antisense Molecules in Epilepsy—A Neuropharmacological Educational Review. Int. J. Epilepsy.

[101] Perucca P., Bahlo M., Berkovic S.F. (2020). The Genetics of Epilepsy. Annu. Rev. Genom. Hum. Genet..

[102] Skotte L., Fadista J., Bybjerg-Grauholm J., Appadurai V., Hildebrand M.S., Hansen T.F., Banasik K., Grove J., Albiñana C., Geller F. (2022). Genome-wide association study of febrile seizures implicates fever response and neuronal excitability genes. Brain.

[103] Berger T.C., Taubøll E., Heuser K. (2022). The potential role of DNA methylation as preventive treatment target of epileptogenesis. Front Cell Neurosci..

[104] Jiang T., Tan M.-S., Tan L., Yu J.-T. (2014). Application of Next-Generation Sequencing Technologies in Neurology. Ann. Transl. Med..

[105] Rădoi V.E., Țurcan M., Maioru O.V., Dan A., Bohîlțea L.C., Dumitrescu E.A., Gheorghe A.S., Stănculeanu D.L., Thodi G., Loukas Y.L. (2023). Homologous Recombination Deficiency Score Determined by Genomic Instability in a Romanian Cohort. Diagnostics.

[106] Myers C.T., Mefford H.C. (2015). Advancing Epilepsy Genetics in the Genomic Era. Genome Med..

